# Alignment of auditory artificial networks with massive individual fMRI brain data leads to generalisable improvements in brain encoding and downstream tasks

**DOI:** 10.1162/imag_a_00525

**Published:** 2025-04-08

**Authors:** Maëlle Freteault, Maximilien Le Clei, Loic Tetrel, Lune Bellec, Nicolas Farrugia

**Affiliations:** Université de Montréal, Montréal, QC, Canada; Centre de Recherche de L’Institut Universitaire de Gériatrie de Montréal, Montréal, QC, Canada; IMT Atlantique, Lab-STICC, UMR CNRS 6285, F-29238, Brest, France; Kitware Europe, Villeurbanne, France

**Keywords:** auditory neuroscience, individual-specific computational models, artificial neural networks, downstream generalisation, deep phenotyping datasets, functional magnetic resonance imaging (fMRI)

## Abstract

Artificial neural networks trained in the field of artificial intelligence (AI) have emerged as key tools to model brain processes, sparking the idea of aligning network representations with brain dynamics to enhance performance on AI tasks. While this concept has gained support in the visual domain, we investigate here the feasibility of creating auditory artificial neural models directly aligned with individual brain activity. This objective raises major computational challenges, as models have to be trained directly with brain data, which is typically collected at a much smaller scale than data used to train AI models. We aimed to answer two key questions: (1) Can brain alignment of auditory models lead to improved brain encoding for novel, previously unseen stimuli? (2) Can brain alignment lead to generalisable representations of auditory signals that are useful for solving a variety of complex auditory tasks? To answer these questions, we relied on two massive datasets: a deep phenotyping dataset from the Courtois neuronal modelling project, where six subjects watched four seasons (36 h) of the*Friends*TV series in functional magnetic resonance imaging and the HEAR benchmark, a large battery of downstream auditory tasks. We fine-tuned SoundNet, a small pretrained convolutional neural network with ~2.5 M parameters. Aligning SoundNet with brain data from three seasons of*Friends*led to substantial improvement in brain encoding in the fourth season, extending beyond auditory and visual cortices. We also observed consistent performance gains on the HEAR benchmark, particularly for tasks with limited training data, where brain-aligned models performed comparably with the best-performing models regardless of size. We finally compared individual and group models, finding that individual models often matched or outperformed group models in both brain encoding and downstream task performance, highlighting the data efficiency of fine-tuning with individual brain data. Our results demonstrate the feasibility of aligning artificial neural network representations with individual brain activity during auditory processing, and suggest that this alignment is particularly beneficial for tasks with limited training data. Future research is needed to establish whether larger models can achieve even better performance and whether the observed gains extend to other tasks, particularly in the context of few-shot learning.

## 1 Introduction

### 1.1 Overall objective

Artificial neural networks (ANNs) have emerged as a powerful tool in cognitive neuroscience. Specifically, ANNs trained to solve complex tasks directly from rich data streams, such as natural images, are able to accurately encode brain activity, that is, predict brain responses directly from the stimulus. A notable observation is that the ANNs which have high performance solving behavioural tasks, for example, image classification, tend to be the ones which perform best to predict brain activity. This was noted first in vision (Yamins et al., 2014) and rigorously established for language while controlling for model architecture and model capacity (Caucheteux et al., 2023). This result suggests that directly training the representations of ANNs to encode well brain activity may lead to more generalisable representations and improved performance on novel downstream tasks. This process, in general called brain alignment (Mineault et al., 2024;Sucholutsky et al., 2023), has only been explored in a few works so far, and most of these works have been carried in the field of vision (Lu et al., 2024;Seeliger et al., 2021;St-Yves et al., 2023) and language (Konkle et al., 2022;Schwartz et al., 2019). These brain alignment works used datasets of limited size, both for brain encoding and downstream tasks. In this work, we explore for the first time the impact of individual brain alignment on an auditory ANN. The study also leverages massive datasets both for training the networks, testing the generalisation of brain encoding to novel stimuli, with the Courtois NeuroMod fMRI dataset (Boyle et al., 2020), and evaluating task performance on a wide range of downstream tasks, with the HEAR benchmark (Turian et al., 2022).

### 1.2 ANNs in audio classification and brain encoding

ANNs trained with deep learning for cognitive neuroscience emerged initially in the field of vision (Schrimpf et al., 2020). CNNs in artificial vision share strong parallels with visual brain processing (Bengio et al., 2013;Krizhevsky et al., 2012). It was found that ANNs trained on complex tasks such as image annotation could predict brain responses to image stimuli with considerable accuracy (Yamins et al., 2014). Building on these foundations, the capability of ANNs for brain encoding has been extended to both language and auditory neuroscience.Kell et al. (2018)designed an auditory CNN with two branches, tailored for music and speech recognition. They discovered distinct auditory processing streams in their network, with the primary auditory cortex best predicted by middle layers, and the secondary auditory cortex by late layers. More recently,Giordano et al. (2023)provided further evidence for the intermediary role of the superior temporal gyrus (STG) in auditory processing using three different CNNs, whileCaucheteux et al. (2023)use of a modified GPT-2 provided new insights into predictive coding theory.

### 1.3 Brain alignment

Typical brain encoding studies re-use a pretrained network based on a very large collection of sounds, and can feature a very large number of parameters. It is relatively straightforward to apply such large networks for brain encoding, for example, using Ridge regression from the latent space of the network to brain activity, as it was done, for example, with BERT (Devlin et al., 2019). Aligning internal representations of ANNs models with brain activity is a much more ambitious goal, which requires directly optimising the parameters of a network in order to maximise the quality of brain encoding through a process called fine-tuning. This optimisation process raises a number of computational and conceptual challenges. First, there is clear evidence of substantial inter-individual differences in functional brain organisation (Gordon et al., 2017;Gratton et al., 2018). For this reason, some authors have advocated for building individual brain models using deep fMRI datasets, where a limited number of individuals get scanned for an extended time, instead of datasets featuring many subjects with limited amounts of data per subject (Naselaris et al., 2021). It is the approach which we decided to take. Second, most fMRI datasets feature only a limited number of stimuli which can be used to train a network. The largest fMRI stimuli sets include Dr Who (approximately 23 h of video stimuli) (Seeliger et al., 2019) and Natural scenes dataset (10k images) (Allen et al., 2022), which is orders of magnitude smaller than what is currently used in the AI field. For example, the latest version of the recent auditory ANN wav2vec (Baevski et al., 2020) has 317 million parameters, and was pretrained with over 60k hours of sound data. It thus seems likely that network architectures should feature less parameters when trained for brain alignment than state-of-art networks trained in the field of artificial intelligence (AI), and the few published studies of brain alignment indeed followed this trend, for example,Seeliger et al. (2021)andSt-Yves et al. (2023).

### 1.4 Brain alignment and generalisation of behaviour

In this work, the term “brain alignment” refers to optimising the parameters of a pretrained ANN to improve brain encoding performance, as outlined in the previous paragraph. In contrast, the term “human alignment,” commonly used in the field of AI safety, refers to ensuring that artificial systems behave in accordance with human intentions, expectations, and benefits (Ji et al., 2023). A recent white paper byMineault et al. (2024)highlights the potential of brain alignment to enhance human alignment in the context of AI safety, through increased robustness of behaviour. This avenue was further discussed at a recent workshop of the 2024 international conference on representational learning (ICLR) (Sucholutsky et al., 2023). However, given the limited size of neuroimaging datasets, compared with datasets commonly used to train AI models, it is not clear that fine-tuning can lead to generalisable performance gains, and may on the contrary distort pre-trained features to overfit training data with poor out-of-distribution performance (Geirhos et al., 2020;Kumar et al., 2022). A few previous studies still found that brain alignment may improve the behaviour of ANNs on downstream tasks (Moussa et al., 2024;Nishida et al., 2020;Palazzo et al., 2020)—tasks with available ground truth to assess performance and that the ANN was not explicitly trained to perform. However, these works examined only a limited number of downstream tasks (one inPalazzo et al. (2020)and four inNishida et al. (2020)andMoussa et al. (2024))and reported, at best, modest performance gains. These limitations may be attributed to the small size of the datasets used for brain alignment or the narrow scope of the downstream tasks considered.

### 1.5 Generalisation scope of brain encoding models

While brain encoding studies are rapidly gaining traction in computational cognitive neuroscience, the scope of generalisation tested in these models remains limited. For instance, seminal work on auditory brain encoding used only 165 sounds, each 2 s long (Kell et al., 2018), and pioneering studies on language comprehension often relied on less than 30 min of recordings per subject, typically featuring a single story (Caucheteux et al., 2023). In vision, the Natural Scene Dataset (Allen et al., 2022) included ~10,000 image stimuli per subject for a visual recognition task, but these images spanned only about 40 semantic categories (Shirakawa et al., 2024). In this study, we aimed to broaden the scale of brain encoding generalisation by using the audio tracks of three full seasons of the*Friends*TV show for training (28 h), and an additional, separate season for testing (9 h). These complex audio stimuli included extensive speech from a large and diverse set of speakers, interwoven with music and a variety of naturalistic sounds. Additionally, we aimed to investigate a broad range of downstream behavioural tasks to assess how brain alignment using*Friends*stimuli influences different aspects of sound processing.

### 1.6 Courtois NeuroMod, HEAR benchmark, and model architecture

In this work, we aimed at demonstrating the feasibility of brain alignment of artificial neural networks in the auditory domain. Specifically, we addressed two general questions: (1) Do brain-aligned networks encode brain activity related to auditory processing more effectively when using novel stimuli? (2) Do brain-aligned networks show improved performance in downstream auditory tasks that are unrelated to the stimuli used for brain encoding?

We made several key design choices to address these two questions:

First, following the approach advocated byNaselaris et al. (2021), we decided to align ANNs at the individual level and compared their performance with ANNs trained at the group level. This allowed us to evaluate whether a smaller but individual-specific dataset is more advantageous than a larger group dataset.

To achieve this, we leveraged the Courtois NeuroMod dataset (Boyle et al., 2020), the largest deep fMRI dataset to date, which was specifically designed by our team to align ANNs with brain data. Its 2022 release features a small number of subjects (N = 6), each with over 100 h of fMRI data, with additional data yet to be released. The dataset spans a wide range of tasks across multiple domains, including several movie-watching datasets with complex soundtracks. For this study, we focused on the*Friends*dataset, which is both extensive (36 h of data per subject) and varied (covering multiple seasons of the TV show with different stimuli in each episode).

Second, in terms of model size and architecture, we selected a pretrained model called SoundNet, which has been shown to perform well in sound processing and brain encoding (Farrugia et al., 2019;Nishida et al., 2020). Soundnet features a limited number of parameters (fewer than 3 millions) as well as a simple convolutional architecture with decreasing temporal resolution, making it well suited to fine-tuning with an fMRI dataset. The*Friends*dataset allowed us to test the generalisation of brain-aligned models to new stimuli in a large controlled distribution (a different season of*Friends*) and to replicate the process of brain alignment with six different subjects.

Third, to assess generalisation abilities on downstream tasks, we leveraged a recent machine learning competition: the Holistic Evaluation of Auditory Representations (HEAR,Turian et al., 2022). HEAR offers a standardised procedure to test the generalisation of the internal representations of a model on a wide array of downstream tasks. Using the HEAR environment, we evaluated our brain-aligned models and, given the large number of teams that participated in the HEAR competition, were able to rigorously compare their performance against a range of state-of-the-art AI approaches.

### 1.7 Study objectives

The specific objectives and hypotheses of our study are as follows:

- Align SoundNet with individual and group brain data and compare the quality of brain encoding with the baseline, non-brain-aligned model. Our hypothesis was that the alignment procedure would lead to substantial gains in brain encoding for within-distribution stimuli drawn from the*Friends*dataset, and that these gains would be subject specific, that is, they would not transfer to other individuals.
- Evaluate how brain alignment impacts out-of-distribution downstream tasks. Our hypothesis was that brain alignment would lead to no degradation or even modest improvements in performance across a wide range of tasks.

Taken together, this study establishes the feasibility and some key methodological decisions to align auditory networks with brain data using massive individual fMRI data, and clarifies how this alignment impacts the performance of networks on downstream tasks (seeFig. 1for an overview of the analysis, including the fMRI data and methodology used to evaluate impact of brain-alignment on both brain encoding and tasks resolution).

**Fig. 1.**
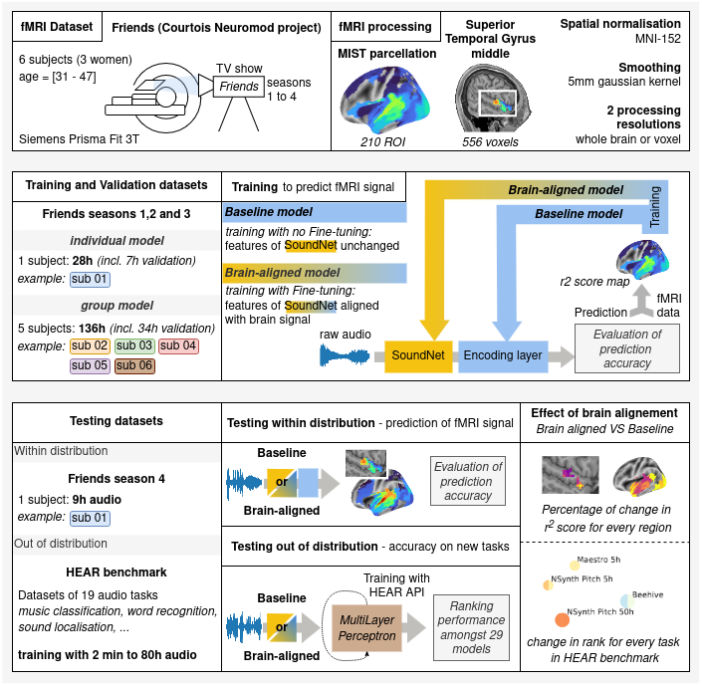
Overview of the analysis. In this study, we used a naturalistic fMRI dataset to align internal features of a pretrained network with brain signals, using an AI training technique called fine-tuning. We evaluated how brain alignment changed the performance of the network, both for tasks that the network has been trained and optimised for (within distribution) and for new tasks (out of distribution).

## 2 Materials and Methods

### 2.1 fMRI data

#### 2.1.1 Participants

Six healthy participants (aged 31 to 47 years at the time of recruitment in 2018), three women (sub-03, sub-04, and sub-06) and three men (sub-01, sub-02, and sub-05) were recruited to participate in the Courtois Neuromod Project for at least 5 years. All subjects provided informed consent to participate in this study, which was approved by the ethics review board of the “CIUSS du centre-sud-de-l’île-de-Montréal” (under number CER VN 18-19-22). Three of the participants reported being native francophone speakers (sub-01, sub-02, and sub-04), one as being a native anglophone (sub-06), and two as bilingual native speakers (sub-03 and sub-05). All participants reported the right hand as being their dominant hand and reported being in good general health. Exclusion criteria included visual or auditory impairments that would prevent participants from seeing and/or hearing stimuli in the scanner and major psychiatric or neurological problems. Standard exclusion criteria for MRI and MEG were also applied. Lastly, given that all stimuli and instructions are presented in English, all participants had to report having an advanced comprehension of the English language for inclusion. The above boilerplate text is taken from the cNeuroMod documentation^*^(Boyle et al., 2020), with the express intention that users should copy and paste this text into their manuscripts unchanged. It was released by the Courtois NeuroMod team under the CC0 license.

#### 2.1.2 Friends dataset

The dataset used in this study is a subset of the 2022-alpha release of the Courtois Neuromod Dataset, called*Friends*, where the participants have been watching the entirety of seasons 1 to 4 of the TV show*Friends*. This subset was selected because it provided a rich naturalistic soundtrack, with both a massive quantity of stimuli and a relative homogeneity in the nature of these stimuli, as the main characters of the series remain the same throughout all seasons. Subjects watched each episode cut in two segments (a/b), also referred as*runs*, to allow more flexible scanning and give participants opportunities for breaks. There is a small overlap between the segments to allow participants to catch up with the storyline. The episodes were retransmitted using an Epson Powerlite L615U projector that casted the video through a waveguide onto a blank screen located in the MRI room, visible to the participants via mirror attached to the head coil. Participants wore MRI-compatible S15 Sensimetric headphone inserts, providing high-quality acoustic stimulation and substantial attenuation of background noise, and wore custom sound protection gear. More details can be found on the Courtois Neuromod project website.^†^

#### 2.1.3 Data acquisition

The participants have been scanned using a Siemens Prisma Fit 3 Tesla, equipped with a 2-channel transmit body coil and a 64-channel receive head/neck coil. Functional MRI data were acquired using an accelerated simultaneous multi-slice, gradient echo-planar imaging sequence (Xu et al., 2013): slice acceleration factor = 4, TR = 1.49 s, TE = 37 ms, flip angle = 52 degrees, voxel size = 2 mm isotropic, 60 slices, acquisition matrix 96 x 96. In each session, a short acquisition (three volumes) with reversed phase encoding direction was run to allow retrospective correction of B0 field inhomogeneity-induced distortion.

To minimise head movement, the participants have been provided individual head cases adapted to the shape of their head. Most imaging in the Courtois Neuromod project is composed solely of functional MRI runs. Periodically, an entire session is dedicated to anatomical scans, see details on the Courtois Neuromod project website.^‡^Two anatomical sessions were used per subject in this study for fMRIprep anatomical alignment, specifically a T1-weighted MPRAGE 3D sagittal sequence (duration 6:38 min, TR = 2.4 s, TE = 2.2 ms, flip angle = 8 degrees, voxel size = 0.8 mm isotropic, R = 2 acceleration) and a T2-weighted FSE (SPACE) 3D sagittal sequence (duration 5:57 min, TR = 3.2 s, TE = 563 ms, voxel size = 0.8 mm isotropic, R = 2 acceleration).

#### 2.1.4 Preprocessing of the fMRI data

All fMRI data from the 2022-alpha release were preprocessed using the fMRIprep pipeline version 20.2.5 (“long-term support”) (Esteban et al., 2019), seeSupplemental File Afor details. We used a volume-based spatial normalisation to standard space (MNI152NLin2009cAsym). The anatomical mask derived from the data preprocessing phase was used to identify and select brain voxels. Voxel-level 2D data matrices (TR x voxels) were generated from 4-dimensional fMRI volumes using the NiftiMasker tool from Nilearn (Abraham et al., 2014) and a mask of the bilateral superior temporal gyri middle (middle STG), specifically parcel 153 and 154 of the MIST parcellation (Urchs et al., 2019), resulting in 556 voxels. ROI-level 2D data matrices (TR x ROI) were generated from 4-dimensional fMRI volumes using the NiftiLabelsMasker tool from Nilearn with the MIST parcellation. The MIST parcellation was used as a hard volumetric functional parcellation because of the availability of anatomical labels for each parcel. This functional brain parcellation was also found to have excellent performance in several ML benchmarks on either functional or structural brain imaging (Dadi et al., 2020;Hahn et al., 2022;Mellema et al., 2022). We chose the 210 resolution of the parcellation atlas because parcels were enforced to be spatially contiguous, and separate regions in the left and right hemisphere. Both the middle STG mask used to select the voxels and the parcels from MIST were based on non-linear alignment. For the voxel-level data matrices, we choose to investigate the effect of spatial smoothing by using BOLD time series with no spatial smoothing or smoothed spatially with a 5 mm gaussian kernel. For ROI-level data matrices, we only used BOLD time series with spatial smoothing (5 mm gaussian kernel). A so-called “Minimal” denoising strategy was used to remove confounds without compromising the temporal degrees of freedom, by regressing out basic motion parameters, mean white matter, and cerebrospinal fluid signals as available in the library load_confounds^§^(equivalent to the default denoising strategy now implemented with load_confounds in Nilearn). This strategy is recommended for data with low levels of motion, as is the case for the Courtois NeuroMod sample (Wang et al., 2023).

### 2.2 Encoding models

#### 2.2.1 Overview

Our approach to training encoding models of auditory activity relied on transfer learning and the fine-tuning of a pretrained deep learning backbone, SoundNet. Audio waveforms served as inputs to the backbone, producing as an output a set of time-dependent features. These features were then used to train a downstream convolutional “encoding” layer to predict the fMRI signal. We explored several training variants, including a baseline where only the encoding layer was trained, as well as fine-tuning experiments where SoundNet parameters were updated up to a certain depth in the network, starting with the final layer (seeFig. 2). Details of the backbone, fMRI encoding layer, hyperparameters, and training procedures are provided below. Models were implemented using PyTorch and other Python^**^libraries and were trained on the Alliance Canada infrastructure with V100 and A100 GPUs.

**Fig. 2.**
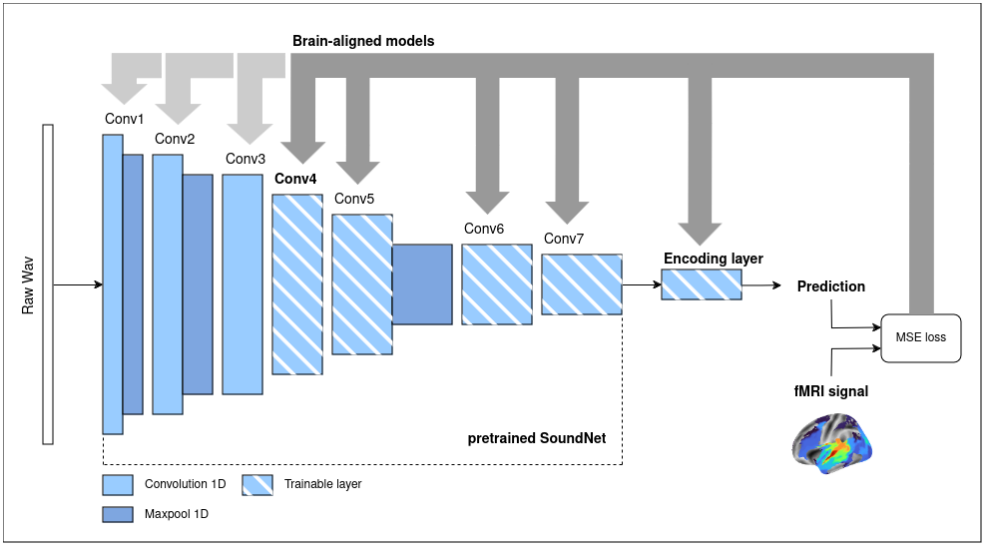
Overview of the training framework. We provided the audio track of the TV show*Friends*to a pretrained convolutional network, SoundNet. Initially, we extracted the output from the 7th convolutional layer of SoundNet, with its parameters*frozen*(fixed values that remain unchanged during training), and used this output as input to train a final encoding layer to predict fMRI activity from a subject watching the TV show. This model serves as our*baseline*. In a second phase, we partially retrained SoundNet along with the encoding layer by fine-tuning all parameters up to the selected layer, allowing these parameters to be updated during training. This new model, where internal layers are fine-tuned to better align with cerebral activity, is referred to as the*brain-aligned*model. The results presented here were obtained using the model fine-tuned up to convolutional layer 4, as depicted in this figure, but we also tested models fine-tuned at various depths, ranging from Conv7 to Conv1.

#### 2.2.2 Deep learning backbone

The network we selected as our backbone is SoundNet, a convolutional neural network with the goal of identifying audio content in an audio excerpt, proposed byAytar et al. (2016). SoundNet was trained to combine information from both the audio and visual inputs of a video, by minimising the Kullback–Leibler divergence (Kullback & Leibler, 1951) between the distribution of its own outputs derived purely from the audio signal, and the output distribution of two different vision networks, obtained with the frames of the video. We selected SoundNet for the following reasons: (1) SoundNet is fully convolutional, as all intermediate representations (i.e., layers) are obtained from 1D convolutions and pooling operators, using directly the audio waveform as input with no additional operations; (2) SoundNet was initially trained on a large dataset of natural videos from the Internet (Flickr), with a high degree of correspondence between the visual and audio content; and (3) SoundNet obtained good performances on downstream auditory tasks using transfer learning, as well as good performance as a brain encoding model (Farrugia et al., 2019;Nishida et al., 2020).

At the time of its release in 2016, SoundNet achieved similar performances to the state-of-the-art (SotA) networks on audio classification benchmarks*Detection and Classification of Acoustic Scenes and Events*DCASE (Mesaros et al., 2017) and*Environmental Sound Classification*ESC-50 (Arandjelovic & Zisserman, 2017;Piczak, 2015). With numerous innovations happening in the AI research field since 2016, as well as the introduction of the dataset AudioSet (Gemmeke et al., 2017), it has since been surpassed by other networks. However, the CNN architecture dominated the leaderboard of many benchmarks up until 2021 for the audio classification task (Gong et al., 2021;Verbitskiy et al., 2022;Wang et al., 2021), and is still quite relevant with the current considerations of the field to find efficient architectures with fewer parameters and reduced energy consumption (Schmid et al., 2023).

While the naturalistic fMRI dataset we used to fine tune the network is the biggest up to date in the fMRI field, the size of the training dataset is still far below what has been offered by larger audio datasets such as AudioSet or VGGSound (Chen et al., 2020;Gemmeke et al., 2017), often used to train the most recent SotA networks. For this reason, we consider that a smaller, simple network was not only necessary in the context of this study, but also beneficial to isolate the impact of brain alignment on network performance. As such, SoundNet provides a generic convolutional network to learn from, with its representations encoding audio features of varying durations, and increasing abstraction in deeper layers.

SoundNet’s architecture (Fig. 2;Table 1) is a series of convolutional blocks that always include the following steps:

**Table 1.** Architecture of the SoundNet network with the two different encoding layers.

**Initial training input size** Audio Input length * TR length (1,49s) * 22,050 Hz						***For sub-03*** : Audio Input length = 70 TR
Layers		Number of out Channels	Kernel size	Stride	Padding	Number of parameters
**SoundNet**						
**Conv1**	Conv1D	16	64	2	32	1,040
	BatchNorm1D	16				32
	ReLU	16				
	MaxPool1D	16	8	8		
**Conv2**	Conv1D	32	32	2	16	16,416
	BatchNorm1D	32				64
	ReLU	32				
	MaxPool1D	32	8	8		
**Conv3**	Conv1D	64	16	2	8	32,832
	BatchNorm1D	64				128
	ReLU	64				
**Conv4**	Conv1D	128	8	2	4	65,664
	BatchNorm1D	128				256
	ReLU	128				
**Conv5**	Conv1D	256	4	2	2	131,328
	BatchNorm1D	256				512
	ReLU	256				
	MaxPool1D	256	4	4		
**Conv6**	Conv1D	512	4	2	2	524,800
	BatchNorm1D	512				1,024
	ReLU	512				
**Conv7**	Conv1D	1,024	4	2	2	2,098,176
	BatchNorm1D	1,024				2,048
	ReLU	1,024				
**Encoding layer whole brain**						
	Conv1D	210	*Kernel size*	1	*Kernel size -1*	1,075,410
**Encoding layer STG**						
	Conv1D	556	*Kernel size*	1	*Kernel size -1*	2,847,276

The values for the number of parameters shown on the right side of the table have been estimated for sub-03 encoding models, with a selected duration of 70 TRs and a kernel size of 5 for both encoding layers. Values vary slightly for each subject (seeTable S1for the different values used for each subject).

- a 1D Convolutional layer
- a 1D Batch normalisation realised on the output of the convolutional layer
- a rectified linear unit function element-wise ReLU.

In some of the blocks, a 1D max pooling is also applied to the output of the preceding steps (seeTable 1for more details).

#### 2.2.3 fMRI encoding layer

We implemented the SoundNet architecture followed by the fMRI encoding layer as a fully end-to-end trainable network (seeTable 1), adapting an open-source PyTorch implementation of SoundNet.^††^Our encoding model predicts entire segments of fMRI data based on corresponding segments of audio waveform data. The SoundNet model is convolutional and non-causal, meaning the output of a filter at a given time point can depend on future time points in the original time series. Accordingly, we designed our encoding layer as a traditional non-causal temporal 1D convolutional layer, with a separate temporal kernel learned for each pair of input features from SoundNet and output features of the brain (either parcel or voxel). The outputs of each feature map after convolution are summed to predict brain activity for a specific brain parcel or voxel. For brain encoding, we used the output of SoundNet’s Conv7 layer, as it matches the temporal resolution of the fMRI signal (0.67 Hz). Formally, the brain encoding layer applies the following model:

$$y_{i}=\sum_{k=0}^{N-1}h_{i,k}⋆x_{k}$$

where

- $y_{i}$is a window of brain activity associated with parcel/voxeli,
- N=2,048is the number of features in layer 7 (andkdenoting a particular feature),
- $h_{i,k}$is a convolution kernel, with varying size and parameters trainable specifically for each pair(i,k),
- $x_{k}$is a temporal window of activity for thek-th feature of layer 7 of SoundNet, and
- ⋆is the valid 1D cross-correlation operator (including padding with a size relative to the kernel size, varying between 6 and 9 s, or 4 and 6 TR).

Note that we explored multiple lengths (from 20 s up to 130 s) for the temporal window which we treated as hyper-parameters for optimisation (seeSupplemental File Bon hyper-parameters exploration).

Notably, with a kernel size of 1, this model is equivalent to a traditional mass-univariate regression of SoundNet features onto brain activity, with no delay between sound waves and brain responses. While the proposed model does not explicitly incorporate an HRF, using a 1D convolution operator with a kernel larger than 1 is analogous to modelling the HRF with a Finite Impulse Response (FIR) filter (Goutte et al., 2000). The temporal window for this FIR filter is determined individually (seeSupplemental File Bon hyper-parameters exploration), with distinct response functions trained for each pair of SoundNet features and brain regions.

However, because the SoundNet features are non-causal, the temporal kernel may also account for differences in the temporal scales of the SoundNet features and fMRI brain activity, rather than solely modelling haemodynamic processes. In other words, the brain encoding layer simultaneously aligns artificial and biological neural processes over time while capturing haemodynamic processes.

#### 2.2.4 Targets for brain alignment

The encoding layer was trained using two different brain targets, depending on what type of fMRI processed data the network learned to predict, thus yielding two different models:

- **STG model:**A model to predict fMRI signal from each 556 voxels located in the STG middle mask, at every TR, resulting in a prediction matrix’s size of 556 voxels prediction by the selected number of TR (seeTable S1for the exact TRs number for every subject). We refer to this model as the*STG model*in the Results section. This model is an evaluation of how well SoundNet predicts auditory fMRI activity in our settings, so we can estimate impact of brain alignment at the voxel level. For training data, we used data with or without spatial smoothing, to evaluate potential effects of spatial smoothing on encoding brain activity.
- **Whole-brain model:**A model to predict the average fMRI signal for all voxels of a parcel at every TR, resulting in a prediction matrix’s size of 210 parcels (also designed as ROI) prediction by the selected number of TR. We refer to this model as the*whole-brain model*in the Results section. The intention for this model is threefold (1) to verify which brain regions can be predicted by the model using audio as an input, (2) to check which ROIs are impacted by brain alignment, and (3) to test whether individual variability has an impact on prediction performance and brain alignment.

#### 2.2.5 Fixed training parameters

To train this architecture, we used AdamW (Loshchilov & Hutter, 2019) as an optimiser for L2 regularisation with weight decay, and we applied a learning rate scheduler that reduces the initial learning rate if no progress is achieved by the optimiser. The weight decay means that the brain encoding layer acts analogously to a Ridge regression, in effect regularising the regression parameters through shrinkage. MSE loss is used to minimise the difference between the predicted and actual fMRI signal.

For training individual models, we used the fMRI data from subjects watching the first three seasons of*Friends*. For each subject, we used 75% of the dataset for training, corresponding to 21 h of training dataset. The remaining 25% was used for validation, corresponding approximately to 7 h of the dataset, and we use all episodes of Season 4 (around 9 h of audio) only for testing (seeTable 2). In addition to individual models, we also trained group models to evaluate whether a greater amount of training data could lead to better prediction results on one subject’s brain activity than using only fMRI data from the same subject. To fairly compare individual and group models, we decided to design a group model specific to each individual model: Unlike individual models where we used fMRI activity from one subject, we used fMRI activity from the other five subjects to train the corresponding group model (around 105 h of dataset used for training, and 34 h). With this method, group models performance will not be influenced by individual features, and only by training dataset size.

**Table 2.** Repartition of the fMRI dataset*Friends*used for individual models between training, validation, and testing.

*Data used for*	**Training**	**Validation**	**Testing**
***Friends***	Seasons 1, 2, 3: 73 episodes		Season 4: 24 episodes
*Percentage*	75%	25%	-
*Number of fMRI runs*	109 (54,5 eps)	37 (18,5 eps)	48 (24 eps)
*Total duration (hours)*	21,37	7,25	9,24

Each episode is split into two halves, with one half shown during an fMRI run.

To evaluate the accuracy of the model’s prediction, we computed the coefficient of determination r^2^between the prediction and the corresponding fMRI time series for each region or voxel, for the entirety of the selected dataset (training or testing).

#### 2.2.6 Hyper-parameters exploration

The goal of this study is to compare performance of an auditory AI network and of the same network but brain aligned. We decided to realise the hyperparameter grid search on the original trained SoundNet, with no fine-tuning, which we will consider as a fixed backbone, also referred to as the baseline model. Through this grid search, we were looking for an optimal set of hyperparameters to ensure SoundNet prediction performance as an encoding model as well as accounting for individual variability in the fMRI dataset. By going through these optimisation steps, we have a better estimation of how much fine-tuning with brain representation impacts a network, for both brain encoding and network performance in classic AI tasks. The selected parameters to be tested only affect the training of the encoding layer at the end of the network.

For each individual model, we optimised different hyperparameters and criteria that could impact the final results (see Supplemental Files B and D ):

- the duration of the audio waveforms given as input in each training iteration,
- the value of the learning rate at the beginning of the training,
- the size of the kernels in the encoding layer,
- the initial weight decay,
- the minimal change value considered for early stopping (referred to as “delta”),
- the number of epochs where such delta change is not present before stopping the training (“patience”).

For the corresponding group model, trained with data from the remaining five subjects, we used the median value of the results from all five individual models.

#### 2.2.7 Fine-tuning the model

While only the encoding layer has been trained in the baseline model, the brain-aligned models have part of the original SoundNet’s parameters retrained to adjust prediction on individual fMRI data. As our architecture can be trained as an end-to-end network, we decided to test different levels of fine-tuning, from training only the last original layer of SoundNet (Conv7) to training the entirety of the network (Conv1). As such, we obtained seven fine-tuned models both for whole-brain and middle STG: Conv1, Conv2, Conv3, Conv4, Conv5, Conv6, and Conv7, each referring to the depth of the network that has been trained. Amongst these seven models, we selected brain-aligned models that have the best ratio between brain encoding performance and training efficiency. We found that models where SoundNet has been fine-tuned up until Conv4 (referred as Conv4 models) achieve the best trade-off (seeSupplemental File E).

#### 2.2.8 Models comparison and statistical analysis for brain encoding

In order to evaluate the encoding performances of the baseline and brain-aligned models, we tried to predict fMRI activity with a null model, using the same architecture as the other models, but with randomly initialised weights. We used a Wilcoxon test (with a threshold of 0.05) to determine whether the difference of the r^2^value of a region/voxel between the null model and the baseline or brain-aligned model was significant across all 48 runs (half-episodes) of*Friends*season 4. As we repeated the same test for 210 regions or 556 voxels, we corrected the p-values obtained through the Wilcoxon test with a false discovery rate (FDR), using the Benjamini–Yekutieli procedure (Benjamini & Yekutieli, 2001), to take in account possible dependencies between tests derived at different regions/voxels. Only significant regions with a false discovery rate*q*inferior to 0.05 were considered as significant. We repeated the same procedure to determine whether the difference of r^2^scores between baseline and fine-tuned models was significant, to evaluate whether fine-tuning SoundNet on brain representations had an impact on SoundNet performances.

#### 2.2.9 Identification and impact of audio annotations in the dataset

To understand the potential effect of brain alignment on the network’s performance, we analysed whether changes in prediction could be driven by specific audio annotations present in the dataset used. Annotations were generated using a ResNet22 network pretrained on AudioSet (Gemmeke et al., 2017;Kong et al., 2020), a dataset including a large diversity of naturalistic audio sounds, ranging from*human voice*to*vehicle*, annotated through 527 labels with different categories and subcategories.

We segmented the audio track from every fMRI run (half episode) in 10 s audio excerpt, and used ResNet 22 to estimate the proportion of audio identified under each label for every excerpt. A subset of Audioset labels was aggregated into eight annotations (seeTable S2for details). For each run, we computed the average proportion of annotations across all 10 s excerpts, giving us in total 48 estimations of every annotation for each season of*Friends*.

To determine whether the different seasons of*Friends*differ qualitatively and quantitatively, we computed a multivariable regression using the ordinary least square method (OLS) to assess whether the proportion of audio labelled under each selected category was significantly different between each season (threshold for significativity set at 0.05). Finally, to evaluate whether the difference in prediction accuracy between the baseline and brain-aligned models could be explained by the presence of specific annotations, we also did an OLS regression for each subject, using the difference in r^2^score (maximum value amongst 210 ROI or 556 voxels) between the baseline and the brain-aligned individual models for each run of Season 4 as our dependant variable, and the difference from the mean proportion of each category for every run as our regressors (threshold for significativity set at 0.05). All statistical analyses have been done using the Python library*Statsmodels*.

### 2.3 Evaluating the models on HEAR

To evaluate how brain alignment impacted SoundNet performances, we tested every brain-aligned and baseline model on the Holistic Evaluation of Audio Representation (HEAR) benchmark (Turian et al., 2022). HEAR was proposed as an AI auditory competition during NeurIPS 2021, and gave the possibility to multiple research teams to test their network architectures and models. This benchmark has been made to evaluate how audio representations from a model are able to generalise over a series of 19 diverse audio tasks, including ESC50 and DCASE 2016, ranging from speech detection to music classification. A wide range of models have been evaluated with this API, resulting in a public ranking of auditory AI models in terms of transfer learning capability at the time of the competition (2022).

As some of the tasks required different inputs, the authors provided an API^‡‡^and preformatted datasets^§§^together with the evaluation code. We followed the API specifications, and extracted the representation of the Conv7 layer to use as scene embeddings for classification/labellisation tasks using the entire audio, and calculated timestamp embeddings (i.e., a sequence of embeddings associated with their occurring times) using the Conv5 layer, for sound event detection or transcription tasks. Both these embeddings are exposed to the HEAR API, which performs the evaluation of all 19 tasks, by using the embeddings as fixed input to train a downstream multi-layer perceptron (MLP). Depending on the task, the final layer could be softmax or a sigmoid, with cross-entropy loss. Details of the hyperparameters for the MLP training can be found in appendix B ofTurian et al.’s (2022)paper.

For each task of the HEAR benchmark, we quantified the change in ranking for the SoundNet model before versus after fine-tuning with brain data, for each subject separately, and for each type of target (Full Brain vs. STG). We applied a Wilcoxon test to determine an overall gain (or loss) in ranking across all the 19 tasks available in HEAR for each configuration separately.

## 3 Results

### 3.1 Comparing sound annotations in the training and test set

We first evaluated to what degree the sound distribution in our training set (*Friends*seasons 1 to 3) matched our test set (*Friends*season 4). For this purpose, we generated annotations of the sounds of each half-episode presented during a single fMRI run using a residual network ResNet 22, pretrained on AudioSet to label audio (Gemmeke et al., 2017; Kong et al., 2020). We further grouped these annotations into meta-categories and only selected the categories where at least 1% of the audio could be recognised within. These include categories such as*Music*,*Laugh*,*Women/Men speak*or*Applause*, with the category*Talking*being the most present amongst all (around 82% through all four seasons, seeFig. 3for details). We then compared the distributions and found few statistically significant differences, and no substantial differences between seasons 1–3 and 4. This result confirms that our generalisation experiment is a large-scale within-distribution generalisation, at least in terms of these high-level categories.

**Fig. 3.**
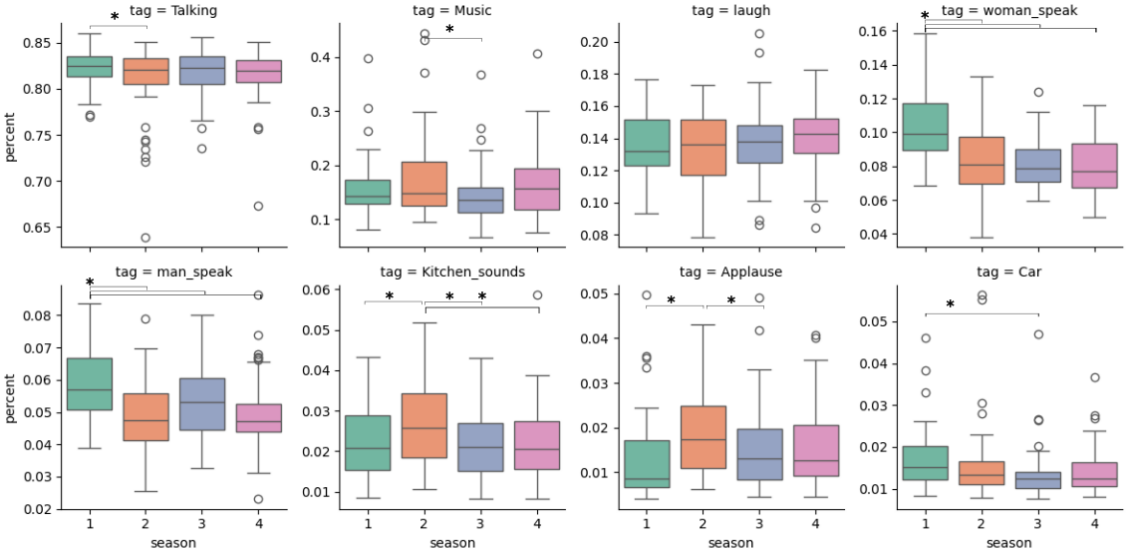
Proportion distribution of labelled audio in all half-episodes between*Friends*’s seasons. The proportion of labelled audio for each half-episode has been obtained using a ResNet 22 pretrained on AudioSet. Pair of seasons with a significantly different distribution in labelled audio proportion are indicated with an asterisk (p < 0.05).

### 3.2 Baseline brain encoding using pretrained SoundNet

#### 3.2.1 SoundNet successfully encodes brain activity in the auditory cortex

**Fig. 4.**
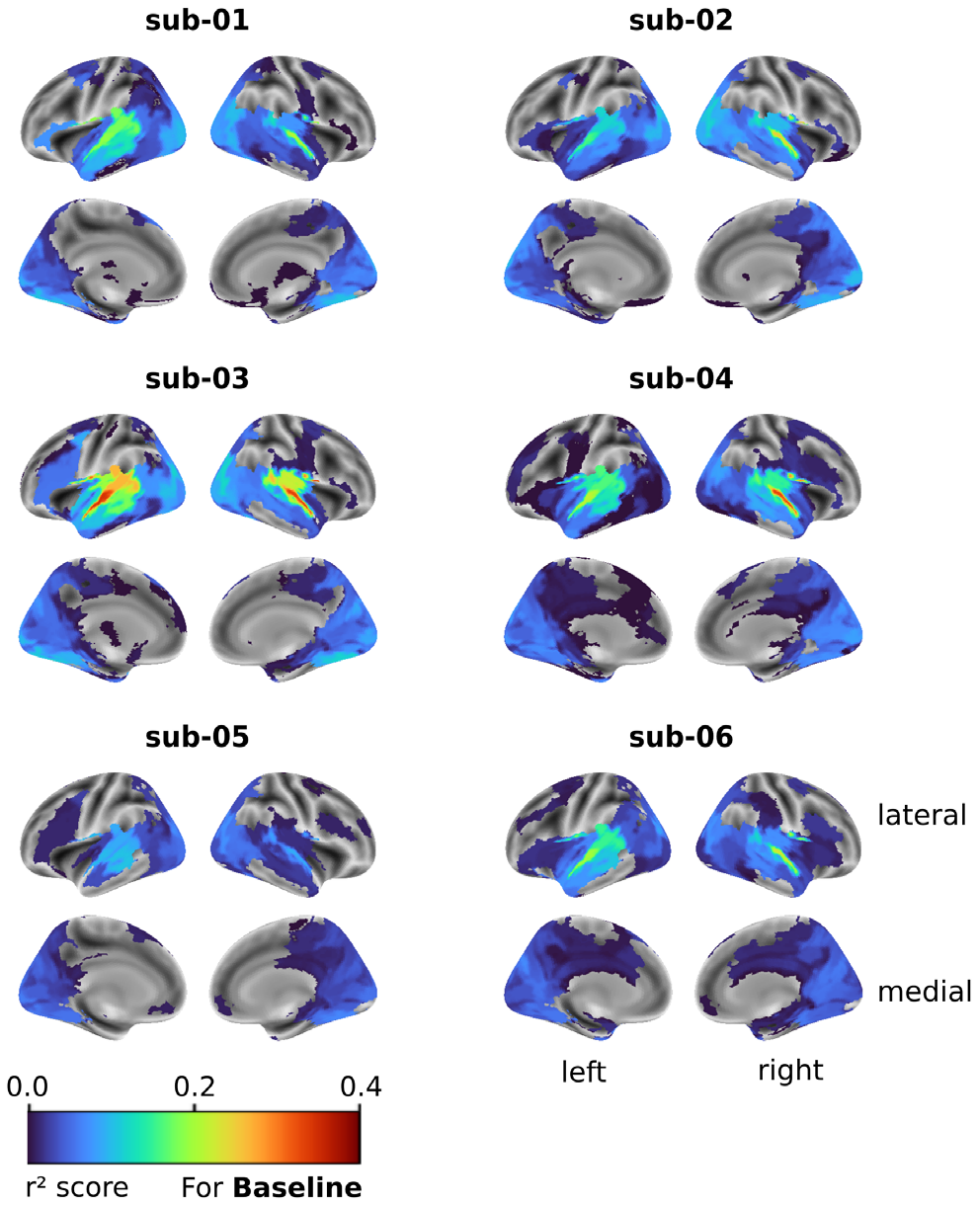
Full brain encoding using SoundNet with no fine-tuning. Surface maps of each subject, showing the r² value for all ROIs from the MIST ROI parcellation. Only parcels with r² values significantly higher than those of a null model initialised with random weights are shown (Wilcoxon test, FDR q < 0.05). Regions with highest r² scores are the STG bilaterally, yet significant brain encoding is achieved throughout most of the cortex, with relatively high values found in the visual cortex as well.

We first tested the ability of our baseline model, SoundNet, to predict fMRI signals in different brain regions, using seasons 1–3 as training and validation and season 4 as test. It performed well, especially in the STG.Figure 4shows almost all subjects had higher r² scores in the middle STG (STGm) than other regions (q < 0.05 for all subjects), except sub-05 whose best predicted parcel was the Middle Temporal Gyrus (MTG) superior. The posterior STG (STGp) also consistently ranked second in terms of prediction accuracy (seeFig. S2).

SoundNet also accurately predicted other auditory regions like the MTG and Heschl’s gyrus in most subjects (Fig. 4). This result supports our hypothesis that our baseline model can encode auditory processing from natural stimuli like movies, with some notable variations in performance between subjects, for example, substantially higher performance was achieved in STG for sub-03 and sub-04.

#### 3.2.2 SoundNet also encodes brain activity in the visual cortex and other regions

Apart from the auditory cortex, brain activity in other regions was also predicted by the models; for most subjects we observed ROIs in the visual cortex such as the Lateral Visual Network DorsoPosterior (LVISnet_DP) and the Ventral Visual Network (VVISnet), respectively, scoring as high as 0.12 and 0.11 (max scores in sub-03). These ROIs proved to be the best predicted ROIs after the STG and the MTG in sub-01, sub-02, sub-05, and sub-06, revealing that our baseline models were also able to encode aspects of the processing of an audio stimulus outside of the auditory cortex.

#### 3.2.3 SoundNet encodes high-resolution brain activity in the superior temporal gyrus

**Fig. 5.**
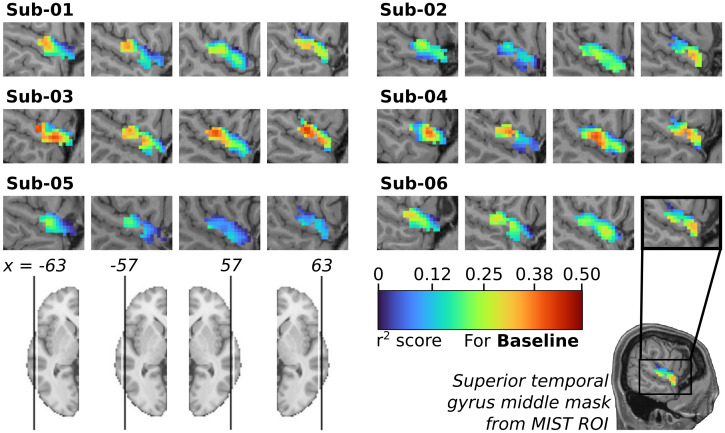
STG encoding using Soundnet with no fine-tuning and fMRI data with spatial smoothing. Mapping of the r² scores from 556 voxels inside the cerebral region defined as the Middle STG by the parcellation MIST ROI, computed by the individual baseline model. To have a better representation of the STG, 4 slices have been selected in each subject, 2 from the left hemisphere (-63 and -57) and 2 from the right hemisphere (63 and 57). Only voxels with r² values significantly higher than those of a null model initialised with random weights are shown (Wilcoxon test, FDR q < 0.05). Individual anatomical T1 has been used as background.

When training the model to predict fMRI time series where a 5 mm gaussian kernel has been applied for spatial smoothing, we found SoundNet could predict fMRI signals from voxels in the middle STG for all subjects (Fig. 5). Most voxels’ fMRI activity was accurately predicted, with r² scores significantly different from the null model (514 to 555 significant voxels out of 556). Subjects 03 and 04 showed the best performance (average max r² of 0.45), while subject 05 performed worse (average max r² of 0.27). These results are consistent with the current literature regarding encoding activity from the auditory cortex and SoundNet ability to encode brain activity in the auditory cortex (Caucheteux et al., 2023;Nishida et al., 2020), and confirm that our model can predict fMRI activity linked to auditory processing at a high spatial resolution. It also shows similar individual differences as observed in full brain encoding. However, when using data with no spatial smoothing, we observe an important decrease in the number of voxels well predicted by the baseline model (seeFig. S3;Section 4).

### 3.3 Fine-tuning SoundNet with individual brain encoding

#### 3.3.1 Individual models do not benefit of the brain alignment the same way

**Fig. 6.**
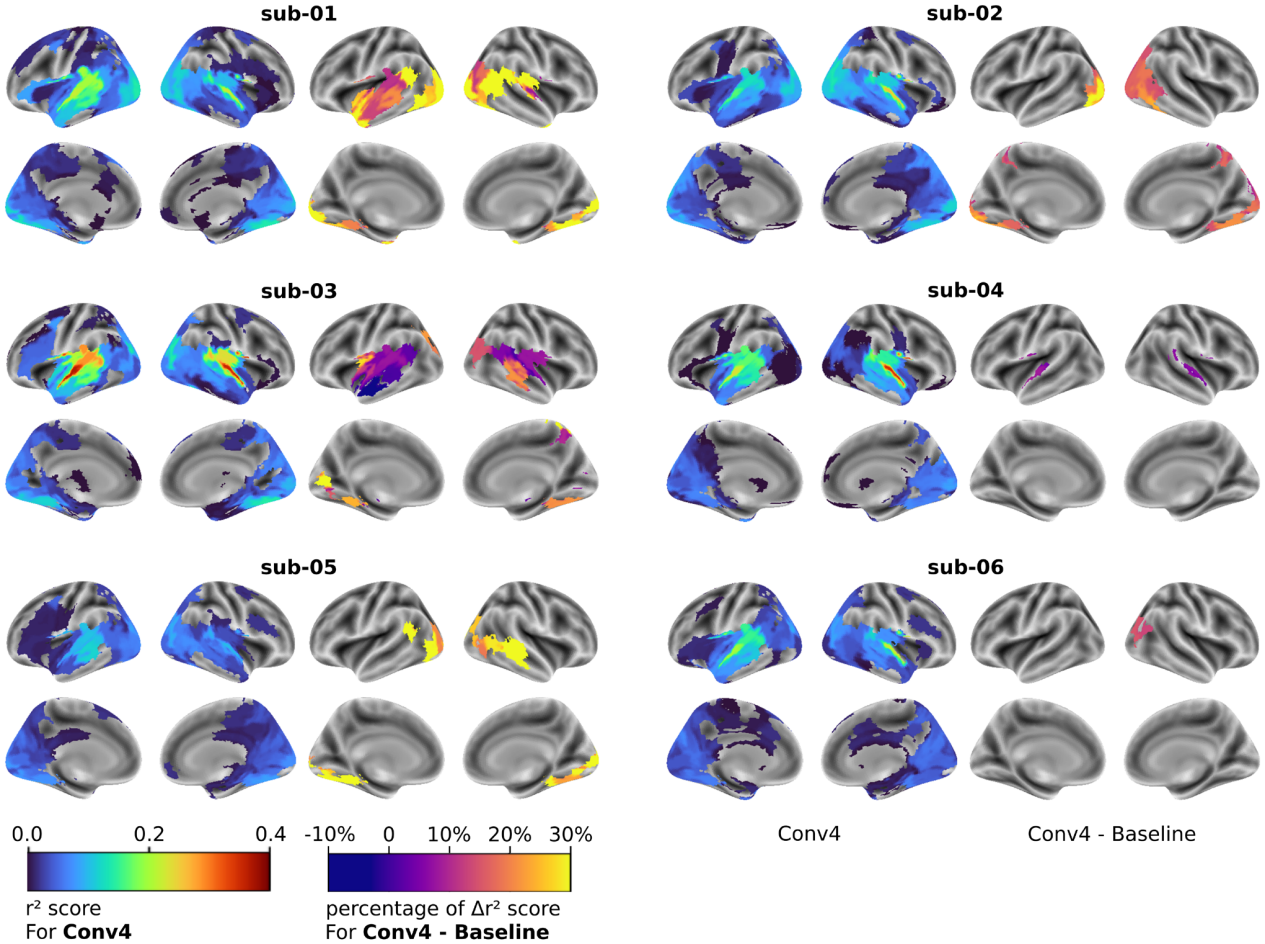
Individual impact of Brain-aligned SoundNet on the full brain encoding. For each subject, on the left side: Surface maps of the r² scores computed with each individual Conv4 model, for the 210 ROIs of the MIST ROI parcellation. Coloured ROIs have an r² score significantly greater than the null model (Wilcoxon test, FDR q < 0.05). On the right side: surface maps of the percentage of difference in r² scores in each ROI between individual Conv4 and baseline models. Only ROIs where Conv4 model have an r² score greater than +/- 0.05 and significantly greater or lesser than the baseline model are displayed (Wilcoxon test, FDR q < 0.05).

We next examined the fine-tuning impact on the brain-aligned models compared with the baseline models. After fine-tuning, the top-predicted parcels for Conv4 models were the same as those at baseline (Fig. 6, left side of each subject panel). For most subjects, STGm and STGp remained the highest-scoring ROIs, with the exception of subject 02 model: while the right STGm is still best predicted, some visual cortices were better encoded than STG regions. We looked at which brain ROIs had the most improvement using Conv4 brain-aligned models.

We tested each ROI’s r² score for significant difference over the baseline and examined both the r² scores difference and the corresponding percentage of difference (seeFig. 6, right side for a brain map of the percentage of r² score difference for each ROI, between the Conv4 and baseline model for each subject): For most subjects, ROIs with the highest improvement in r² score gained between 0.01 and 0.04, with a relative gain from their original value of 15% to 30%, depending on the ROI and the subject. However, sub-05 brain-aligned model, whose baseline model had the worse r² scores amongst all subjects, showed the highest relative gain in the MTG posterior (+ 0,03 r² score, corresponding to a gain of 167% of the original value). In general, ROIs with low r² scores (between 0.05 and 0.15) showed higher relative improvement than ROI with high r² score. The fine-tuning’s main improvements were not always in the auditory cortex: while for subjects 01, 04, and 05, the highest gain in r² score was in the right STG or MTG (between +0.02 and 0.04 r² score), for the remaining subjects, it was located in the ventral, lateral, or posterior visual network. Overall, fine-tuning improved the quality of brain encoding overall, with substantial variations across subjects in terms of both the magnitude and location of improvements.

#### 3.3.2 Fine-tuning at the voxel level also leads to substantial improvements in brain encoding

**Fig. 7.**
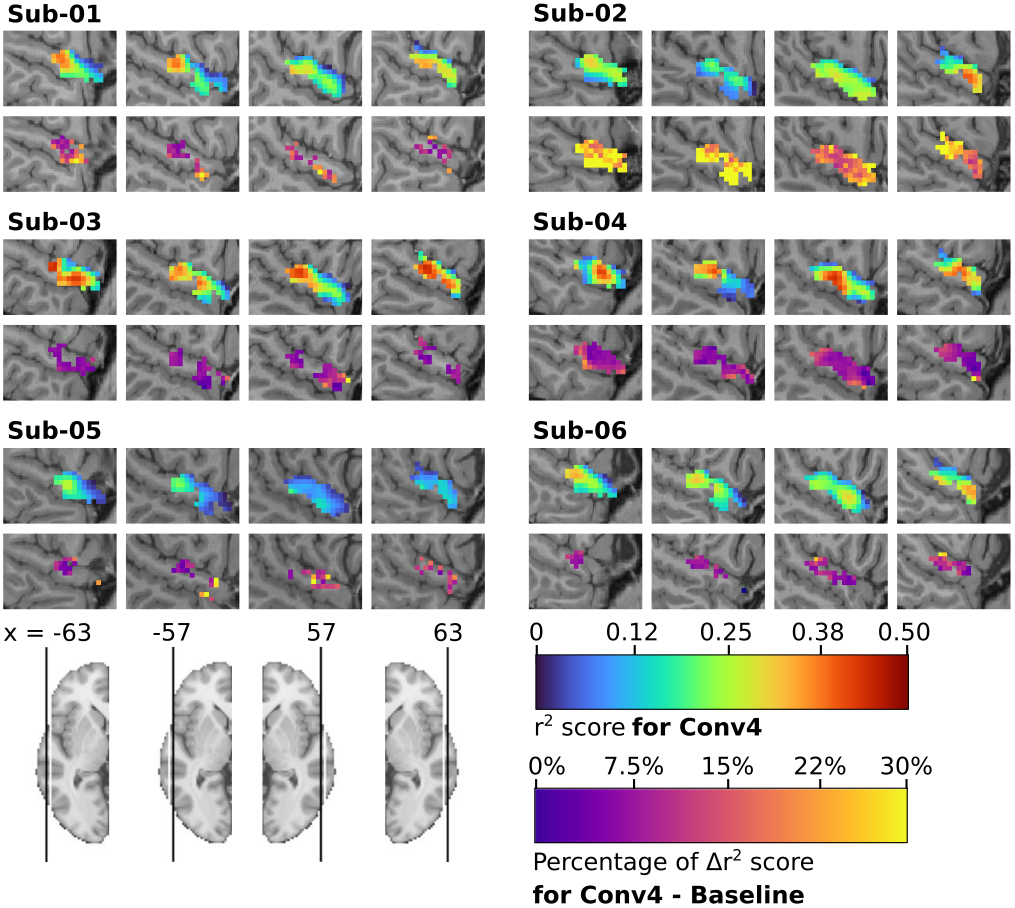
STG encoding using brain-aligned SoundNet and fMRI data with spatial smoothing. For each subject, on the top: mapping of the r² scores from 556 voxels inside the cerebral region defined as the Middle STG by the parcellation MIST ROI, computed by the individual Conv4 model. Only voxels with r² values significantly higher than those of a null model initialised with random weights are shown (Wilcoxon test, FDR q < 0.05). For each subject, on the bottom: mapping of the difference of r² scores between the Conv4 model and the baseline model. Only voxels from the Conv4 model with r² values greater than +/- 0.05 and significantly greater or lesser than those of the baseline model are shown (Wilcoxon test, FDR q < 0.05). Individual anatomical T1 has been used as background.

We next wanted to see whether fine-tuning also affected voxel-level fMRI signal predictions. We calculated the r² score difference between the baseline and the brain-aligned Conv4 model for each voxel in the STGm ROI, and only mapped those with significant differences (Fig. 7). For both cases of training data (whether with or without spatial smoothing), we found voxels that were well predicted by the baseline models also had the most significant r² score increases for all subjects, although voxels with lower prediction accuracy were also impacted by fine-tuning. However, when using spatial smoothing, the median gain across all voxels was between 7% and 26% depending on the subject, lower than relative gains found in the brain-aligned models fine-tuned on the whole brain. While there were far less voxels being encoded when using data without spatial smoothing, the median gain in r2 score was higher for most subjects, between 10% and 115% (seeFig. S4). Overall, we found that fine-tuning improved brain encoding at the voxel level, with marked variations across subjects, and some departure from the impact of fine-tuning at the level of the full brain.

#### 3.3.3 Improvement in prediction in brain-aligned models does not associate strongly with specific audio features

To investigate some of the potential reasons that could lead to the prediction improvement observed with the brain-aligned model, we searched for correlation between the presence of specific features in the audio of season 4 and the prediction change. Using a residual network ResNet 22 pretrained on AudioSet to label the audio content, we computed the proportion of audio related to 28 categories for every half-episode of season 4 of Friends. With the categories where at least 1% of the audio of season 4 could be associated with, we computed a multivariable regression using the ordinary least square method, to explain the difference in r² score for each half-episode in season 4. While tendencies can be found for categories such as Talking, Kitchen sounds, or Car, no tag shows a significant impact through every model. This analysis did not reveal any major influence of the selected features on the prediction amelioration made by the brain-aligned models, but points to individual models being differently affected by the features, seeFigure S5in Supplemental File H.

#### 3.3.4 Brain-aligned individual models are subject specific, but group models show similar performance to individual models

**Fig. 8.**
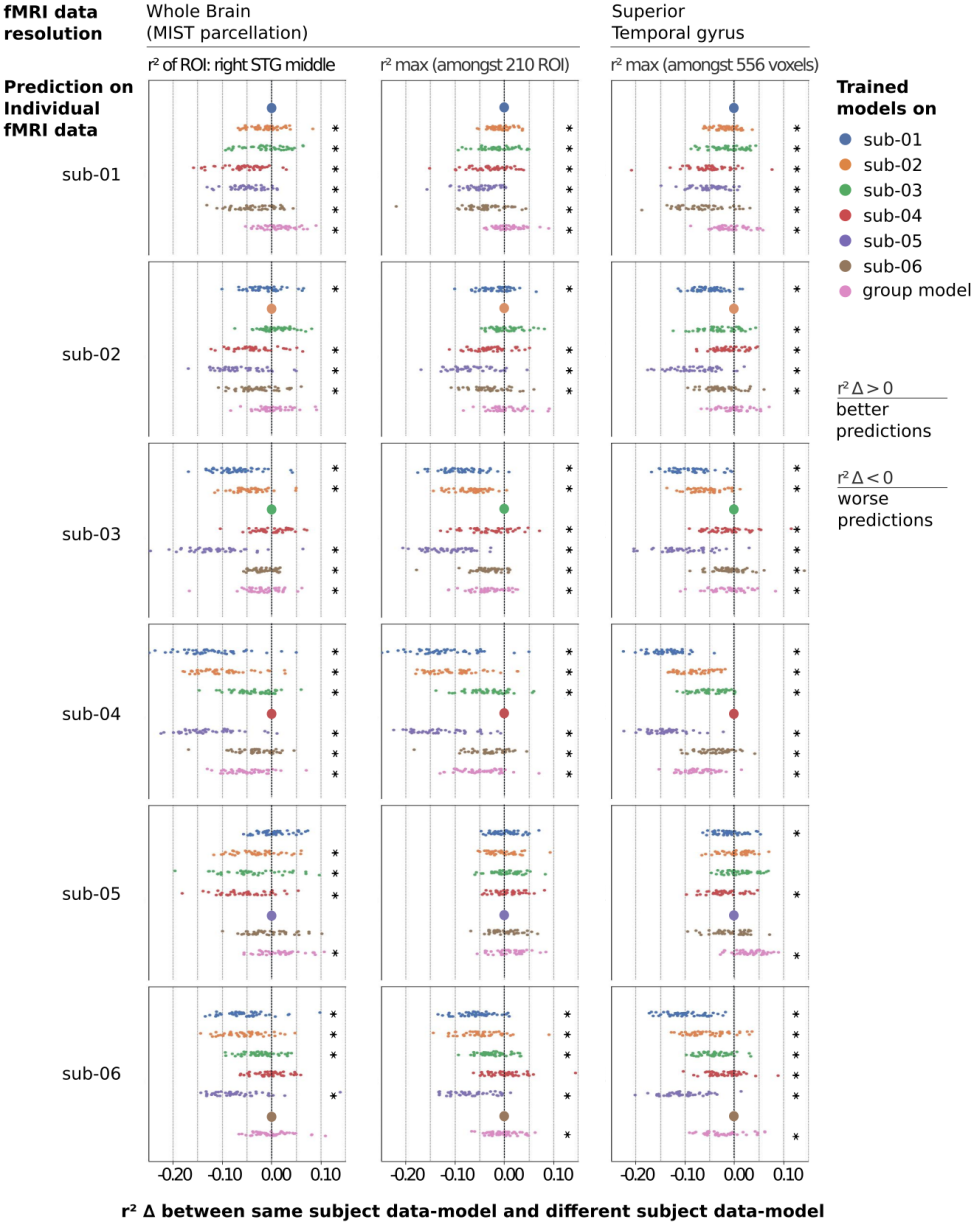
Comparison of prediction accuracy for subject-specific fMRI data using models trained on the same versus other subjects’ data. We computed the difference of r² scores computed by a brain-aligned model trained on data from the same subject as the test data, versus trained on one (from blue to brown) or a group of individual data (pink) different from the subject’s data used for testing. The difference is computed for each of the 48 half-episodes of the fourth season of*Friends*. A Wilcoxon test has been used to determine whether the difference was significant between one individual model and the group model as well as each of the other five individual models (p < 0.05).

Finally, we evaluated whether the fine-tuned models were subject specific, by applying models trained on data from one subject to fMRI signals collected on other subjects. When evaluating the difference for the maximal r² score in all ROI (respectively, all voxels) in the whole-brain model (respectively, STG model), we found that the model trained on one specific subject had the best performance to predict this specific subject’s data, both the whole-brain and middle STG models, with the exception of sub-05 (Fig. 8). When looking at the difference in right STG middle ROI, sub-05 results are similar to other subjects. Overall, trained models appeared to exhibit subject-specific features.

Results for group models show larger variability. For whole-brain models, two group models significantly outperform their respective individual models, two perform significantly worse, and the remaining two show no significant differences. For STG models, while four out of six individual models still significantly outperform their group counterparts, the group model performance often approaches that of the best encoding model.

Although the sample size is too small for definitive conclusions, it is noteworthy that subjects who benefited from a group model approach tended to have low baseline brain encoding performance.

### 3.4 Fine-tuning improves SoundNet ranking in diverse AI auditory benchmarks

We aimed to evaluate the impact of brain alignment on the performance of SoundNet with downstream tasks, using the HEAR benchmark. For each task in the benchmark, we ranked both brain-aligned models and Soundnet amongst the 29 other models tested with this benchmark, and compared brain-aligned models against SoundNet. We analysed the difference of performance from three different angles:

- Between individuals and group models (12 models by task), including both whole-brain and middle STG, to evaluate whether training on a larger dataset is more advantageous than training on individual dataset (seeFig. 9),
- Between whole-brain and middle STG models (six models by task), to evaluate whether the resolution of the training dataset could influence changes in performance in different tasks (seeFig. 10),
- Between each individual subject (seeFig. 11), to see whether individual features also have different effects depending on the task.

#### 3.4.1 Brain-aligned individual and group models display higher generalisation than SoundNet

After evaluating SoundNet in each task using the HEAR API, we found that SoundNet did not perform well in most tasks, being part of the least performing models multiple times. Brain-aligned models performed significantly better than SoundNet (p < 0.05) in 12 tasks out of 19, and performed worst in 2 tasks (DCASE 2016 and VoxLingua107 top 10) (seeFig. 9andFig. S6for more details). Gain in performance due to brain alignment is not related to a specific type of audio task, as brain-aligned models perform better in a variety of tasks, as shown inFigure 10. When taking both middle STG and whole-brain models into account, we did not find any significant difference between individual and group models, both showing an average gain of two ranks.

**Fig. 9.**
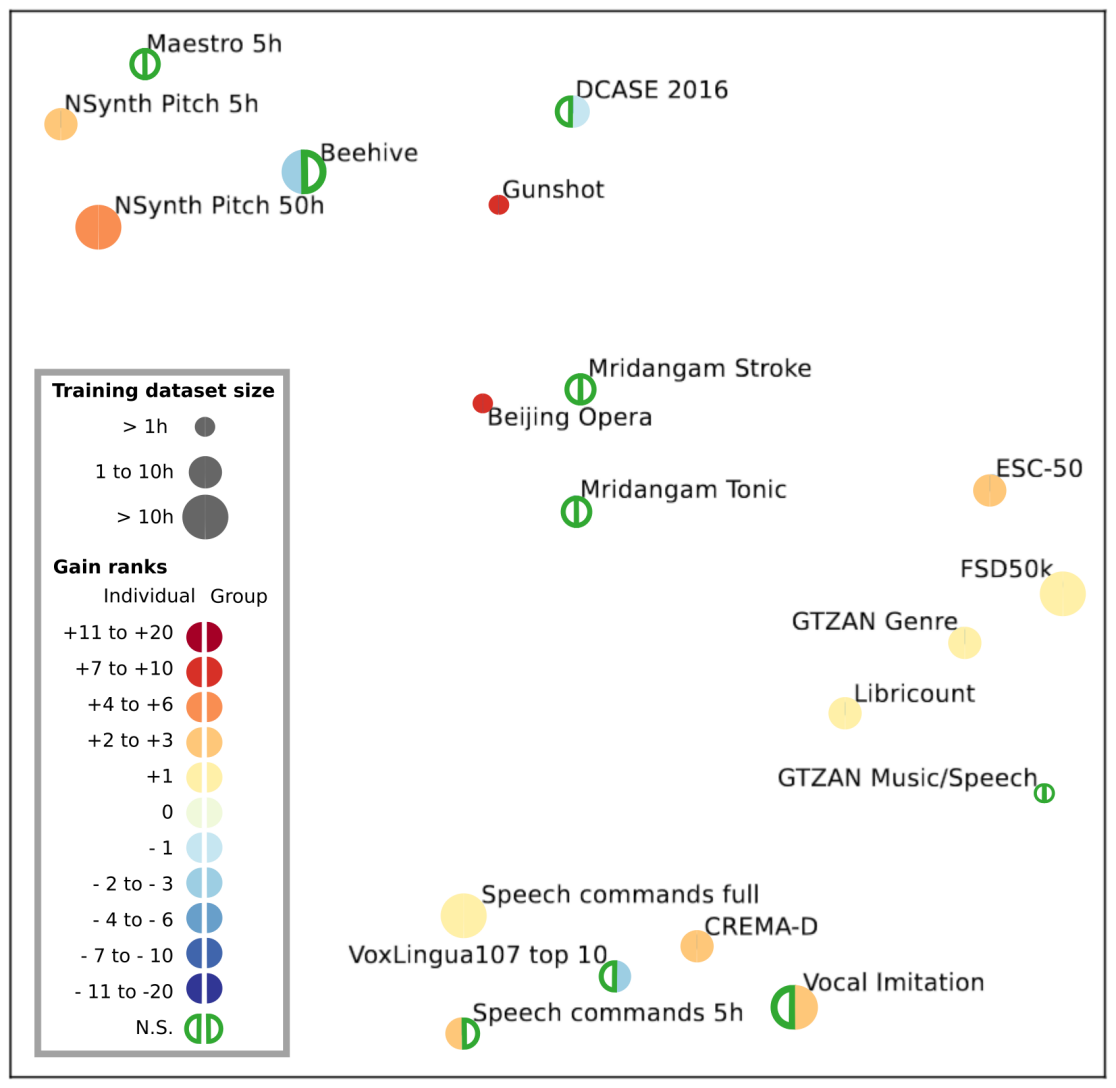
Rank variation between Conv4 and baseline models on all tasks from the HEAR benchmark. Adaptation offigure 2of appendix B from the original HEAR paper (Turian et al., 2022), showing a similarity visualisation of all 19 tasks, based upon normalised scores. For each task, the change of rank between the baseline model and the Conv4 model is symbolised by a coloured circle. Performance from both whole-brain and STG versions of the individual models (half-circle on the left) and group models (half-circle on the right) has been averaged for each of the 19 tasks from the HEAR benchmark. When the change of rank is equal to +1 (light yellow), Conv4 model is performing better than SoundNet at the task, but does not outperform other models. Significativity has been tested using a Wilcoxon test (p < 0.05).

**Fig. 10.**
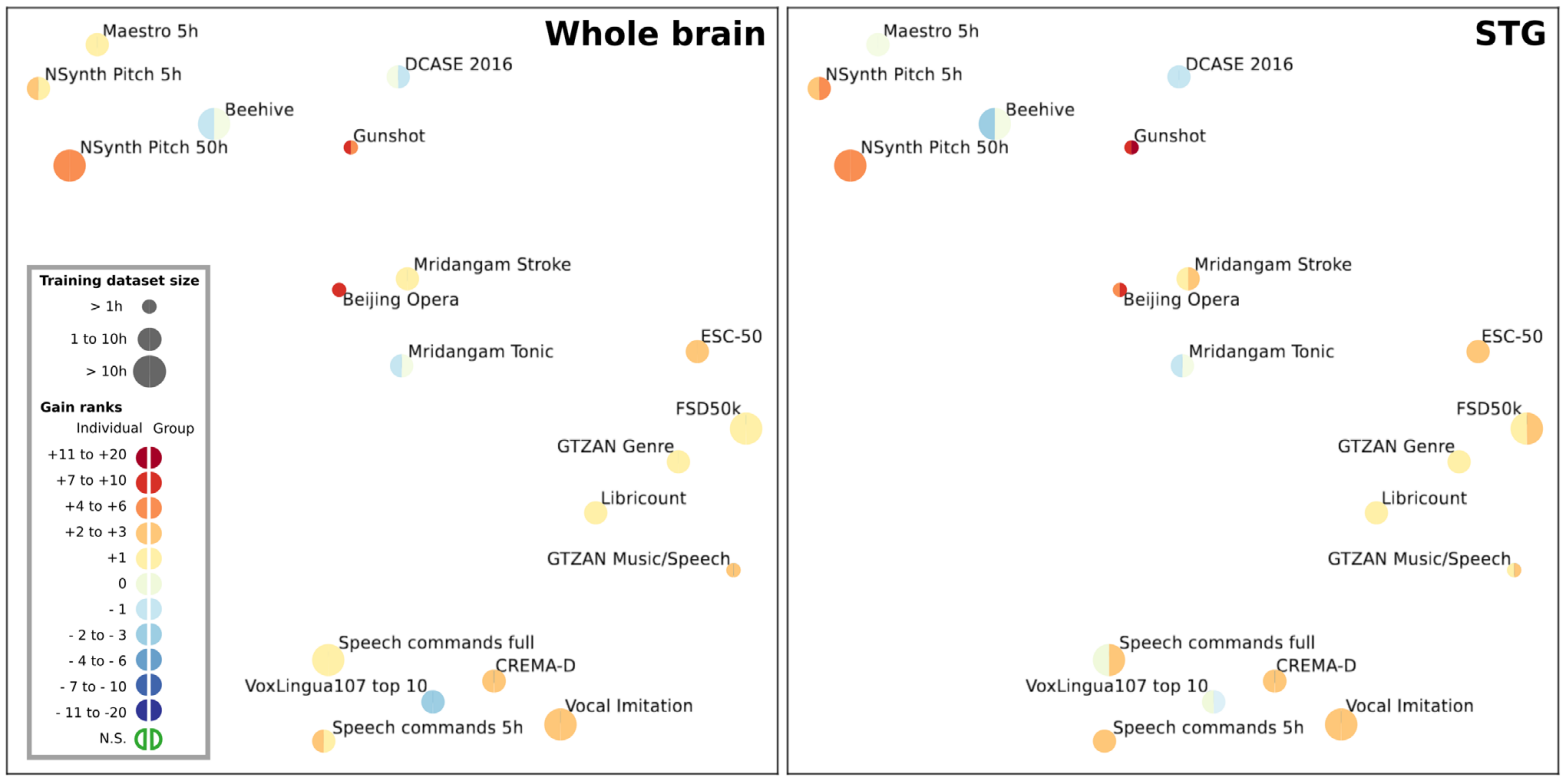
Rank variation between whole-brain and middle STG models on all tasks from the HEAR benchmark. Adaptation offigure 2of appendix B from the original HEAR paper (Turian et al., 2022), showing a similarity visualisation of all 19 tasks, based upon normalised scores. For each task, the change of rank between the baseline model and the Conv4 model is symbolised by a coloured circle. Left: Average change of rank with the whole-brain models (six models for half a circle). Right: Average change of rank with the STG models (six models for half a circle). Due to the low number of models per task, significance for each task has not been tested at this level.

We also observed that the brain alignment led to important gains in rank for a few tasks, such as Gunshot Triangulation, Beijing Opera, and the NSynth Pitch (5 and 50 h). Brain alignment seems to have the biggest impact with tasks involving small training datasets: For Gunshot Triangulation, brain-aligned models surpassed up to 18 other models, while they only had 2 min to retrain the parameters necessary to solve the task. Beijing opera shows similar results, but also has the highest standard deviation in ranking amongst all tasks. Considering that most models tested in this task scored around 0.95 in accuracy, we consider that the change in ranks observed for Beijing Opera is highly affected by the ranking distribution (seeFig. S6). In summary, brain alignment increases the generalisation capability of SoundNet.

#### 3.4.2 Both whole-brain and voxel-level fine-tuning result in better generalisation, but training on bigger datasets improves performance for voxel-level models

Taking both individual and group models together, the comparison between whole-brain models and STG models yielded no significant differences in performance. Both showed an average improvement of two ranks across all tasks compared with SoundNet, with the highest rank gains observed in the same tasks: Gunshot and Beijing Opera.

However, when analysing separately the performance of group models and individual models, we found an influence of the resolution of the training dataset. At the voxel level, group models significantly outperformed individual STG models (p < 0.05), with an average rank gain for all tasks of 2.4 and 1.7, respectively. However, no significant differences were observed between individual and group models trained on the whole brain.

#### 3.4.3 Performance of individual models varies between subject and task

**Fig. 11.**
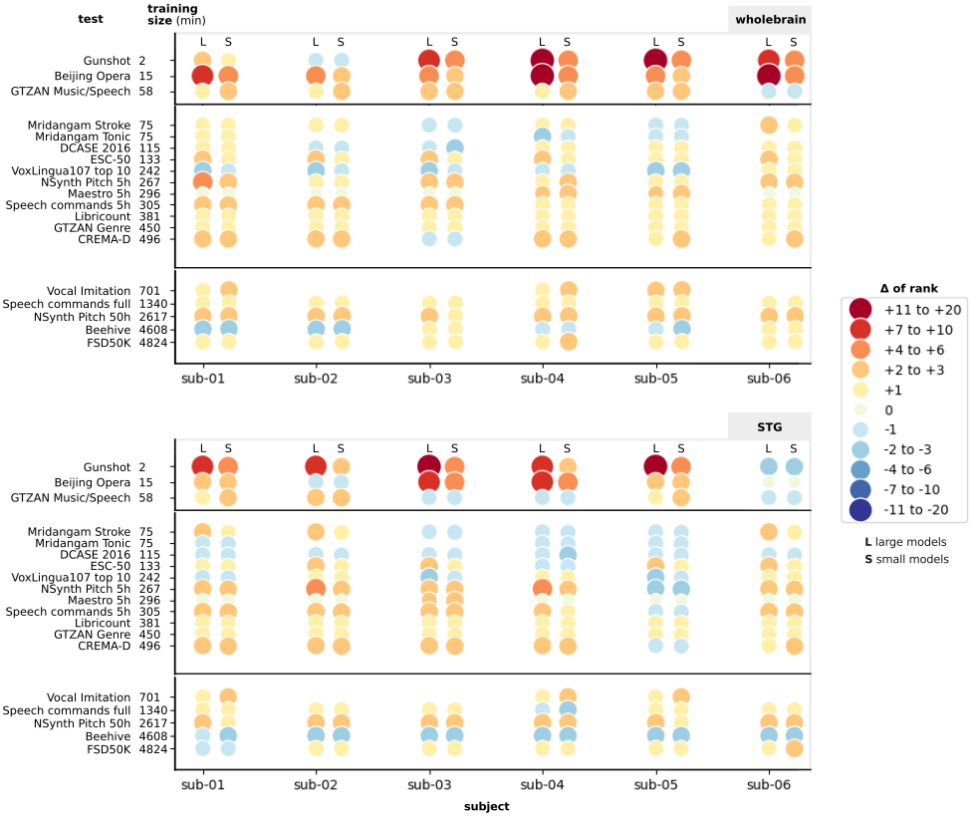
Rank variation between Conv4 and baseline models on all tasks from the HEAR benchmark, ordered by dataset size. Each individual Conv4 model (both whole-brain and middle STG models) has been used to resolve the 19 tasks from the HEAR benchmark, ordered by the size of training dataset available through the benchmark. We extracted from the official HEAR Benchmark Leaderboard^***^the performances of 8 small models (up to 12 M parameters) and 21 large models (from 22 to 1,339 M parameters). We compared our brain-aligned and baseline models performances against the ones from large models (L columns, on the right side for each subject), and small models (S columns, on the left side for each subject). For each task, the change of rank between the baseline model and the Conv4 model is symbolised by a coloured circle.

For the individual models, we wanted to compare brain-aligned models performance with that of models of similar size (around 3 millions parameters). To do so, we divided the 29 models in 2 evaluation groups, depending on the number of parameters: a first group of 8 small models having less than 12 millions parameters, and a group with the remaining 21 models, ranging from 22 to 1,339 million parameters for the larger models (Fig. 11).

Using whole-brain fine-tuning, all six individual brain-aligned models displayed a significant gain in rank in the benchmark (over SoundNet and other models) (p < 0.05) (left panel ofFig. 11). When comparing how brain-aligned models ranked amongst large models and small models (respectively,*L*column and*S*column, for each individual model inFig. 11), results were similar: all individual models had a significant increase in gain rank amongst small models, and five individual models displayed a similar increase amongst large models, with the exception of whole-brain sub-2 model, where its increase was still close to be significantly different (p = 0.057).

Results with individual middle STG models were more heterogeneous, with only sub-01, sub-02, and sub-03 Conv4 models ranking significantly higher than SoundNet, amongst small models and large models (right panel ofFig. 11).

Overall, all individual models show gains in rank in at least half of the tasks compared with SoundNet, but tasks with better performance are not the same through all individual models, showing important individual variability.

## 4 Discussion

In this study, we explored the benefits of aligning a pretrained auditory neural network (SoundNet) with individual brain activity, in terms of both generalisation of brain encoding to new types of stimuli and behavioural performance on a wide range of downstream tasks. Our results confirm substantial improvements in encoding brain activity, with gains extending beyond the auditory cortex, for example, in the visual cortex. Importantly, brain alignment led to significant enhancements in performance across a broad range of auditory tasks when assessed using transfer learning. Our study also highlighted notable inter-individual variations, in terms of both the impact of brain alignment on brain encoding quality and in the performance gains for downstream tasks.

### 4.1 Can task-optimised ANN be aligned with brain activity?

Our findings suggest task-optimised ANNs can successfully be aligned with individual brain activity. While we observed substantial enhancements in brain encoding quality, the extent of these improvements varied across both brain regions and individuals.

When models were directly fine-tuned to encode voxel-level STG activity with spatial smoothing, all participants exhibited modest but significant improvement in the superior temporal gyrus (STG) in most voxels. However, using data without spatial smoothing has reduced significantly the number of voxels encoded by both baseline and brain-aligned models, for all subjects. This result could be explained by a smaller tSNR at lower resolution, compared with data with spatial smoothing (Molloy et al., 2014). Considering the voxel size (2 mm isotropic), a 5 mm gaussian kernel can be considered as a good middle point between loss of spatial resolution and loss of tSNR. We also note that even without spatial smoothing, fine-tuning the models with fMRI data still improves the accuracy of the prediction in the few voxels that were well encoded in the baseline.

When models were fine-tuned on the entire brain, the STG remained the best predicted region in the brain after brain alignment. However, the impact of this process varied across both brain regions and individuals. For most subjects, regions outside the STG such as the visual cortex experienced improvements comparable or greater to those in the STG. Different reasons can explain this result: Activation of the visual cortex by auditory stimuli has been observed in different contexts (Cate et al., 2009;Wu et al., 2007): Research in multimodal processing of spoken language found that visual cortices seem to be part of this specific process (Seydell-Greenwald et al., 2023;Van Atteveldt et al., 2004), and considering that the subjects were watching an audiovisual stimuli with a relative high amount of language content (around 80%), finding activation in the visual cortex in this context is coherent with the literature. To add up to this, the best predicted region in our study is the Superior Temporal Gyrus middle, always followed by the Superior Temporal Gyrus posterior and the Middle Temporal Gyrus, instead of the Heschl’s gyrus (primary auditory cortex). These regions have also been shown to play a key role in the integration of visual and auditory information in the brain (Beauchamp et al., 2004;Proverbio et al., 2011;Van Atteveldt et al., 2004). Our results seem to indicate that our models have effectively learned the multimodal processing of audio stimuli within a naturalistic context, where the visual cortex is also involved. As the individual brain-aligned models are highly specific of the individual data used for the training, it is also possible that these results directly reflect specificity of individual processing learned with the brain alignment. However, it could also be due to the correlation of audio and visual features in our video stimuli (for instance, the presence of faces and lip movements during speech). It is challenging to draw direct comparisons with previous studies due to the sensitivity of the R2 metric to data acquisition and preprocessing decisions, including smoothing and voxel resolution.

### 4.2 Impact of specific audio annotations on model’s performance

Further research is needed to understand the sources of variability in performance between individual models and to clarify which aspects of tasks and brain activity are critical for benefiting from brain alignment. Using a ResNet22 to annotate the audio content of Season 4 of*Friends*, we investigated potential audio features that might influence results. While no single feature consistently impacted all models, we observed tendencies indicating that individual models were influenced differently and weakly by various audio features. This observation is consistent with our downstream tasks (see below), which almost uniformly benefitted from brain alignment. Benefits of brain alignment thus do not appear to be limited to narrow categories of sound stimuli.

A limitation of this work is the temporal scale used for annotations: we averaged annotation results over half an episode, where multiple scenes with different audio contexts (e.g., kitchen, café, outdoors) occur together. Currently, we lack annotations related to the visual content of*Friends*episodes, which prevents us from encoding brain activity at the scene level. Investigating the correlation between brain encoding performance and audio annotations at the scene level, rather than over half an episode, would be an important step in further exploring these tendencies.

### 4.3 Task performance on downstream tasks

We evaluated the performance of our brain-aligned models against SoundNet using the HEAR benchmark, which encompasses a variety of auditory tasks, and found that brain alignment generally benefited performance on downstream tasks. Few studies have employed a downstream task benchmark after brain alignment.Palazzo et al. (2020)reported modest performance gains in vision tasks post-alignment with EEG data.Nishida et al. (2020)reported similar findings with their audiovisual tasks, using fMRI, as well asMoussa et al. (2024)for semantic tasks. However,Schwartz et al. (2019)reported no significant change in performance after brain alignment with fMRI or MEG data. Our research differs notably in stimulus nature (a TV show), and the very large volume of fMRI data used for fine-tuning. While our results seem to align with the first three studies in terms of finding mostly moderate improvements in performance, this study is the first to examine a wider range of auditory downstream tasks. The primary goal of the HEAR benchmark is to evaluate the capacity of a network’s internal representations to generalise to new tasks with data of a different nature than what has been used to initially train the network. Considering this goal, brain aligning a pretrained CNN network led to more generalisable representations, but also identify possibly large gains for downstream tasks with limited training data available: The two tasks that benefited the most are gunshot triangulation (a classification task) and Beijing Opera percussion (an instrument recognition task), which are small scale datasets (Turian et al., 2022) (training data correspond to approximately 100 and 900 s, respectively). However, our results also show improvements on much larger datasets, such as NSynth pitch classification on 5 and 50 h of data (Engel et al., 2017), as well as modest benefits on a very large and difficult benchmark, FSD50k, a multi-label audio tagging dataset with more than 80 h of training data (Fonseca et al., 2021). Taken together, the ability of our brain-aligned representations to generalise to small and larger scale datasets suggests both that they are general enough to generalise with few data and flexible enough to enable gains on larger scale tasks.

### 4.4 Do models benefit more from individual datasets, compared with bigger datasets?

We compared performance of ANNs trained on individual datasets versus trained on bigger group datasets for both brain encoding (within distribution) and tasks from the HEAR benchmark (out of distribution).

For models trained to predict STG fMRI activity at the voxel level, we observed that four out of six individual models significantly outperformed group models trained on multiple subjects in predicting fMRI activity for the same subject. Amongst the remaining two, the group model for sub-05 performed better than its corresponding individual STG model. Notably, the sub-05 individual model was the weakest performer amongst all individual models, and the sub-05 dataset had the lowest temporal signal-to-noise ratio (tSNR) of all datasets, seeSupplemental File Bfor more details. This suggests that, at the voxel level, having data with a high tSNR may be crucial for an individual model to capture subject-specific processing and outperform group models in predicting fMRI activity. However, it remains unclear whether individual specificity aids generalisation to new tasks, as individual STG models performed worse than their respective group STG models on these tasks.

For whole-brain models, the benefits of individual models compared with group models remain unclear. While two individual models significantly outperformed their group counterparts in encoding brain activity, two performed worse, and no significant differences were observed for the remaining two. Additionally, group models do not show improved generalisation, as individual and group models perform similarly on many downstream tasks. Given the current focus in the AI field on reducing computational costs and the challenges of acquiring sufficiently large fMRI datasets for training neural networks, this work suggests that smaller, individual fMRI datasets can be as effective as larger datasets in improving network generalisability for whole-brain models.

### 4.5 Longer temporal windows for training led to better encoding results

At a technical level, hyper-parameter optimisation was an important step for effective fine-tuning of SoundNet. We adopted an approach that combines multiple steps, starting with an extensive grid search on one subject, and then refining the optimal parameters per subject on a subset of the grid and parameters that had the most notable impact in our initial investigation (seeSupplementary Results 1). A surprising finding was that the optimal duration of the input window for sound waves extended up to 70 TRs (105 s). Two main factors may explain this observation. First, it is known that auto-regressive models of fMRI activity improve their performance even for very context windows. Our group recently published a study using the*Friends*dataset where we found the best model to use 286 s (4 min and 46 s) of fMRI data to predict the next time point (Paugam et al., 2024). There is thus evidence of long-term memory processes in fMRI brain data. It is possible that these processes reflect in part exogenous stimuli such as sound. Another possibility is that SoundNet was trained to generate visual annotations from the sound of short videos. It is thus intrinsically biassed towards the duration of these videos, ranging from a few seconds to a few minutes. In any case, a take-away of our study is that brain alignment is sensitive to hyper-parameter optimisation and this work may provide a guide for selecting ranges and parameters in future works.

### 4.6 Problems of a within-distribution testing dataset for brain encoding

The annotation study showed that the TV show used in the dataset for this study presents strong similarities between each season, resulting in within-distribution training and testing. This kind of training may possibly lack in diversity to pretrain an ANN and check for broad generalisation of representations. The CNeuroMod data collection includes a variety of stimuli beyond the*Friends*TV show, and it would be possible to check how different types of stimuli impact generalisation to downstream AI tasks. Additionally, we are interested in investigating whether brain-aligned models lead to human-like similarity judgement patterns (Bakker et al., 2022).

### 4.7 Limitations

A limitation of this study is to focus on a single pretrained network, Soundnet. Considering recent advances in AI auditory models performance (Schmid et al., 2023), it would be important to study other architectures as well in the future, to evaluate whether and how brain alignment impact could differ depending on the architecture used (e.g., transformers vs. convolutional networks), the number of model parameters, and the type of data used for pretraining. We also found that SoundNet had a lower score in benchmarks such as ESC-50 and DCase, compared with the scores of its original paper. While we tried to stay as close as the original implementation, multiple reasons could explain this difference of score: it is possible that the original SoundNet paper used different embeddings to evaluate the model, compared with the embedding required by the HEAR benchmark. We also used Python with Pytorch to implement the brain-aligned models and end-to-end training, while originally SoundNet was done in Lua with Tensorflow. The conversion from one library to another could also have an impact.

It should finally be noted that the parcels used from the MIST ROI parcellation were based on non-linear alignment; while the models trained on the whole-brain fMRI activity best predicted the STG, they also displayed important individual differences. We cannot exclude the possibility that specific ROIs in the auditory cortex and the visual cortex could be slightly misaligned with individual anatomy, which could partially impact the results.

### 4.8 Conclusions

In our study, we developed the first set of auditory deep artificial networks fine-tuned to align with individual participants’ brain activity. This was made possible by the Courtois NeuroMod project’s massive individual data collection effort. We successfully fine-tuned a pretrained network called SoundNet to better encode individual participants’ brain signals, showing varying degrees of improvement over a model that only adds an encoding layer to predict brain signals. These brain-aligned models also improved in performance a pretrained network, trained without brain data on a diverse set of AI audio tasks, ranging from classifying pitch to determining the number of speakers. The brain-aligned models also demonstrate high potential for tasks with limited dataset available and few-shot learning. These findings open many avenues for future research, ranging from studying inter-individual variations to testing brain alignment for various model architectures, types of training data, and types of downstream tasks.

## Supplementary Material

Supplementary Material

## Data Availability

The fMRI data used to train the model are openly available through registered access at linkhttps://www.cneuromod.ca/access/access/. All the code used to train the models, produce the results and figures is freely accessible on a github repository athttps://github.com/brain-bzh/cNeuromod_encoding_2020.

## Footnotes

*
https://docs.cneuromod.ca/en/latest/index.html

†
https://docs.cneuromod.ca/en/latest/index.html

‡
https://docs.cneuromod.ca/en/latest/index.html

§
https://github.com/SIMEXP/load_confounds

**
https://github.com/brain-bzh/cNeuromod_encoding_2020

††
https://github.com/smallflyingpig/SoundNet_Pytorch

‡‡
https://hearbenchmark.com/hear-api.html

§§
https://hearbenchmark.com/hear-tasks.html

***
https://hearbenchmark.com/hear-leaderboard.html
