# Supplementary material for: Alignment of auditory artificial networks with massive individual fMRI brain data leads to generalisable improvements in brain encoding and downstream tasks
Source: Imaging Neurosci (Camb). 2025 Apr 8;3:imag_a_00525. doi: 10.1162/imag_a_00525 (PMC12319826; doi:10.1162/imag_a_00525)
Supplement: Supplementary Material [file imag_a_00525-supp.pdf]

Supplemental material

Supplemental - Methods

Supplemental File A - fMRIprep workflow

Results included in this manuscript come from preprocessing performed using *fMRIPrep* 20.2.5 (@fmriprep1; @fmriprep2; RRID:SCR_016216), which is based on *Nipype* 1.6.1 (@nipype1; @nipype2; RRID:SCR_002502).

A total of 2 T1-weighted (T1w) images were found within the input BIDS dataset for each subject. Anatomical preprocessing was reused from previously existing derivative objects.
For each of the BOLD runs found per subject (across all tasks and sessions), the following preprocessing was performed. First, a reference volume and its skull-stripped version were generated by aligning and averaging 1 single-band reference (SBRefs). A B0-nonuniformity map (or fieldmap) was estimated based on two (or more) echo-planar imaging (EPI) references with opposing phase-encoding directions, with 3dQwarp @afni (AFNI 20160207). Based on the estimated susceptibility distortion, a corrected EPI (echo-planar imaging) reference was calculated for a more accurate co-registration with the anatomical reference. The BOLD reference was then co-registered to the T1w reference using bbregister (FreeSurfer) which implements boundary-based registration [@bbr]. Co-registration was configured with six degrees of freedom. Head-motion parameters with respect to the BOLD reference (transformation matrices, and six corresponding rotation and translation parameters) are estimated before any spatiotemporal filtering using mcflirt [FSL 5.0.9, @mcflirt]. First, a reference volume and its skull-stripped version were generated using a custom methodology of fMRIPrep. The BOLD time-series were resampled onto the following surfaces (FreeSurfer reconstruction nomenclature): fsaverage. The BOLD time-series (including slice-timing correction when applied) were resampled onto their original, native space by applying a single, composite transform to correct for head-motion and susceptibility distortions. These resampled BOLD time-series will be referred to as preprocessed BOLD in original space, or just

preprocessed BOLD. The BOLD time-series were resampled into standard space, generating a preprocessed BOLD run in MNI152NLin2009cAsym space. First, a reference volume and its skull-stripped version were generated using a custom methodology of fMRIPrep. Grayordinates files [@hcppipelines] containing 91k samples were also generated using the highest-resolution fsaverage as intermediate standardised surface space. Several confounding time-series were calculated based on the preprocessed BOLD: framewise displacement (FD), DVARS and three region-wise global signals. FD was computed using two formulations following Power (absolute sum of relative motions, @power_fd_dvars) and Jenkinson (relative root mean square displacement between affines, @mcflirt). FD and DVARS are calculated for each functional run, both using their implementations in Nipype [following the definitions by @power_fd_dvars]. The three global signals are extracted within the CSF, the WM, and the Whole-brain masks. Additionally, a set of physiological regressors were extracted to allow for component-based noise correction [CompCor, @compcor]. Principal components are estimated after high-pass filtering the preprocessed BOLD time-series (using a discrete cosine filter with 128s cut-off) for the two CompCor variants: temporal (tCompCor) and anatomical (aCompCor). tCompCor components are then calculated from the top 2% variable voxels within the brain mask. For aCompCor, three probabilistic masks (CSF, WM and combined CSF+WM) are generated in anatomical space. The implementation differs from that of Behzadi et al. (2007) in that instead of eroding the masks by 2 pixels on BOLD space, the aCompCor masks are subtracted a mask of pixels that likely contain a volume fraction of GM. This mask is obtained by dilating a GM mask extracted from the FreeSurfer's aseg segmentation, and it ensures components are not extracted from voxels containing a minimal fraction of GM. Finally, these masks are resampled into BOLD space and binarized by thresholding at 0.99 (as in the original implementation). Components are also calculated separately within the WM and CSF masks. For each CompCor decomposition, the k components with the largest singular values are retained, such that the retained components' time series are sufficient to explain 50 percent of variance across the nuisance mask (CSF, WM, combined, or temporal). The remaining components are dropped from consideration. The head-motion estimates calculated in the correction step were also placed within the corresponding confounds file. The confound time series derived from head motion estimates and global signals were expanded with

the inclusion of temporal derivatives and quadratic terms for each [@confounds_satterthwaite_2013]. Frames that exceeded a threshold of 0.5 mm FD or 1.5 standardised DVARS were annotated as motion outliers. All resamplings can be performed with a single interpolation step by composing all the pertinent transformations (i.e. head-motion transform matrices, susceptibility distortion correction when available, and co-registrations to anatomical and output spaces). Gridded (volumetric) resamplings were performed using antsApplyTransforms (ANTs), configured with Lanczos interpolation to minimize the smoothing effects of other kernels [@lanczos]. Non-gridded (surface) resamplings were performed using mri_vol2surf (FreeSurfer).

Many internal operations of *fMRIPrep* use *Nilearn* 0.6.2 [@nilearn, RRID:SCR_001362], mostly within the functional processing workflow. For more details of the pipeline, see the section corresponding to workflows in *fMRIPrep*'s documentation[1].

The above boilerplate text was automatically generated by fMRIPrep with the express intention that users should copy and paste this text into their manuscripts *unchanged*. It is released under the CC0 license

[1] https://fmriprep.org/en/latest/workflows.html

Supplemental File B - Exploration of Hyperparameters space

Sub	Audio input length (tr)	Initial learning rate	Kernel size	Weight decay	*Early Stopping* Patience (epochs)	Delta
03	1	1.10^{-2}	1	1.10^{-2}	10	**0**
	10	1.10^{-3}	3	**1.10^{-3}**	**15**	0,1
	30	**1.10^{-4}**	**5**	1.10^{-4}	20	0,5
	**70**		9			
04	60	1.10^{-3}	4			
	70	1.10^{-4}	5			
	**80**	**1.10^{-5}**	**6**	**1.10^{-3}**	**15**	**0**
06	60	1.10^{-3}	4			
	**70**	1.10^{-4}	5			
	80	**1.10^{-5}**	**6**	**1.10^{-3}**	**15**	**0**
01	60	1.10^{-4}	5			
	70	**1.10^{-5}**	6			
	**80**	1.10^{-6}	**7**	**1.10^{-3}**	**15**	**0**
02	60	1.10^{-4}	5			
	70	**1.10^{-5}**	6			
	**80**	1.10^{-6}	**7**	**1.10^{-3}**	**15**	**0**
05	**60**	1.10^{-4}	5			
	70	1.10^{-5}	6			
	80	**1.10^{-6}**	**7**	**1.10^{-3}**	**15**	**0**

Table S1. Hyperparameters values explored for subjects' baseline model. The values selected for fine-tuning the models are marked in bold. The subject order has been constrained by the availability of all four season fMRI data for each subject at the time of the analysis, as this study was taking place in parallel to the data collection. For the kernel size and the learning rate, the range of values shifted by 1 unit between sub-06 and sub-01, as the optimal values for sub-4 and sub-06 appeared to be the maximal value of the explored window, and we wanted to ensure optimal hyperparameters for each subject.

As we are working on individual datasets, we wanted to explore the hyperparameters space for each subject's baseline models. However, doing so would be particularly time-consuming and highly costly in terms of computational resources. In order to constrain the hyperparameters grid search, we decided to explore all parameters only with the baseline model trained on one subject fMRI dataset. We chose to use Sub-03, as their time series had the highest temporal Signal-to-Noise Ratio (tSNR) and lowest motion levels amongst all 6 subjects (Boyle et al., 2023). The tSNR has been averaged in the brain mask of each run of Friends seasons 1 and 2, as well as two others datasets of the Courtois Neuromod project. The tSNR has been extracted by MRIQC, using the MRIQC API (Esteban et al., 2017).
We trained the baseline model (fixed-weights SoundNet + encoding layer) to predict fMRI activity from sub-03, with 1296 different configurations of hyperparameters (see Table 3 for the selected values explored for each criteria). This step has been done twice, for sub-03 STG model and Whole-brain model, but as results were equivalent between both, we decided to only keep results from the Whole-brain model. To distinguish between all configurations of hyperparameters, we ranked trained models by their configuration and their prediction performance on the validation set: we used the r^2 score of the best predicted parcel as our measure or performance. We computed a correlation matrix between ROI predictions from the best 100 configurations, to determine if some of the models trained on these configurations shared a similar parcel prediction pattern. We observed 2 to 3 clusters of configurations, so in order to better define these clusters, we used an agglomerative hierarchical clustering, with a linkage function computing the Euclidean distance between centroid of clusters, and divided the output in 3 clusters (UPGMC algorithm, as implemented by the Scipy library[2]). For each cluster, we computed the

[2]https://docs.scipy.org/doc/scipy/reference/generated/scipy.cluster.hierarchy.linkage.html#scipy.cluster.hierarchy.linkage

corresponding predicted brain map by averaging the prediction output of all configurations in the cluster. We then compared all 3 clusters, and chose the cluster with the highest maximal r^2 score (best predicted parcel amongst 210) and highest mean r^2 score (mean of all 210 r^2 scores). When looking at the values for each hyperparameter, we determined which value was prevalent in the best cluster, by computing the statistical mode on the values selected for all configurations present in this cluster (see Supplementary Result 1).

After deciding which parameters and values had the most impact on the capacities of the baseline model to predict fMRI signals from sub-03 dataset, we switched to other subjects, this time exploring only the most impactful hyperparameters on sub-03 and a limited set of values around the optimal value found for sub-03 (see Table 3). As a result, we explored only 27 configurations for each of the remaining subjects. We defined the best hyperparameters values by computing the mode amongst the 10 most performing configurations. We did not use cluster analysis for other subjects, as we only had 27 configurations. As the data collection was done in parallel to the hyperparameter grid-search, we started this process with data from a few subjects (sub-03, sub-04 and sub-06). After selecting the hyperparameters configuration for both sub-06 and sub-04, we decided to adjust the window of tested values of two hyperparameters for the remaining subjects, as their optimal values seemed to always be the highest value for both sub-06 and sub-04 (see Supplemental Table 3 for more details).

Supplemental File C - Details of AudioSet categories used for audio annotation

Talking (81.66%) *15 Audioset labels:*
Speech, Hubbub / speech noise / speech babble, Male speech / man speaking, Female speech / woman speaking, Child speech / kid speaking, Conversation, Narration / monologue, Babbling, Children shouting, Screaming, Whispering, Laughter, Baby laughter, Giggle, Baby cry / infant cry

Music (16.17%) *1 Audioset label:*
Music

Laugh (13.70%) *1 Audioset label:*
Laughter

Woman speak (8.72%) *1 Audioset label:*
Female speech / woman speaking

Man speak (5.22%) *1 Audioset label:*
Male speech / man speaking

Kitchen sounds (2.32%) *11 Audioset labels:*
Door, Cupboard open or close, Drawer open or close, Dishes pots and pans, Cutlery / silverware, Chopping (food), Sink (filling or washing), Water tap, faucet, Kettle whistle, Microwave oven, Blender

Applause (1.59%) *2 Audioset labels:*
Applause, Cheering

Car (1.52%) *6 Audioset labels:*
Car passing by, Tire squeal, Motor vehicle (road), Car, Bus, Vehicle

Table S2. Categories used to label audio features in the *Friends* dataset. The categories are derived from AudioSet labels. Categories are in descending order, from Categorie most present in the audio of the dataset to least present (mean percentage through all 4 seasons, from top to bottom, left to right column).

Supplemental - Results

Supplemental File D - Optimal hyperparameters substantially improve brain encoding with SoundNet

We optimised the hyperparameters for training our brain encoding model, which predicts individual brain activity using SoundNet internal representations. Through a systematic grid search, we explored the impact of several key hyperparameters, including learning rate, temporal window size, convolutional kernel size, weight decay, and early stopping parameters.

We tested 1296 configurations of hyperparameters on the fMRI data from sub-03. When ordering each configuration by the maximal r^2 score obtained on the validation set for the Whole-brain baseline model, we observed an important gap between the first and last models: models with best r^2 scores scored as high as 0.39, when models ranking 400 and below achieved a r^2 score of 0.07 at best. A similar result has been found for the middle STG baseline model, with a maximal r^2 score of 0.45, and a decrease of the score up to 0.05 for the worst performing models. This gap has also been observed when clustering the 100 best models. The agglomerative clustering of the r^2 brain encoding maps using the centroid method with Euclidean distance computed 3 clusters of configurations for the Whole-brain baseline model: the maximal r^2 score in the averaged predicted brain map of the best cluster, with 20 configurations, is 0.36, and the mean r2 score is 0.033, while for the second cluster, the scores drop respectfully to 0.29 and 0.013 (70 configurations), and to 5.10^{-2} and 8.10-4 for the last cluster (10 configurations).

Through these experiments, we identified an optimal set of hyperparameters that were then used for fine-tuning the models. Overall, our hyperparameter optimization process played a crucial role in enhancing the quality of brain encoding and selecting the most effective hyperparameter configuration for the subsequent analyses.

Supplemental File E - Conv4 fine-tuning leads to substantial improvements in brain encoding

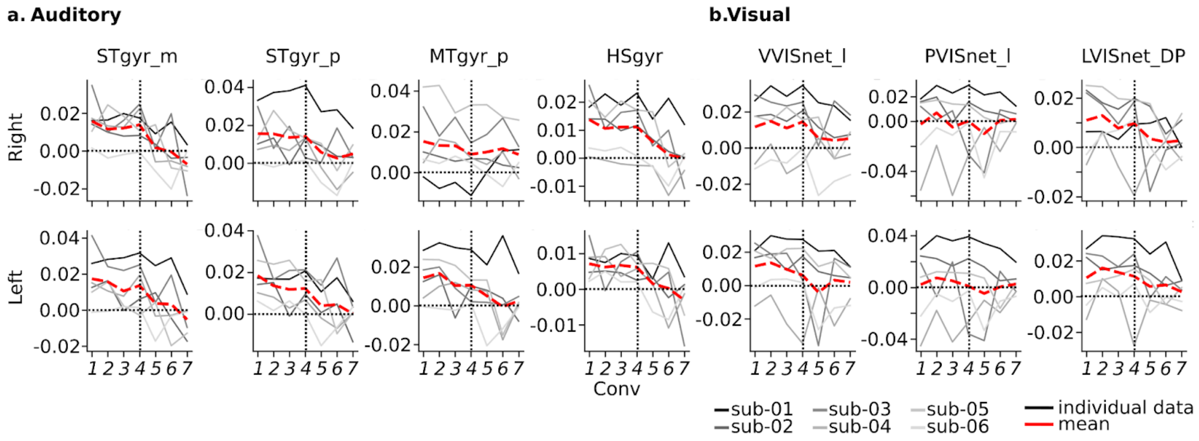

Figure S1. Impact of fine-tuning internal layers in SoundNet on individual brain encoding. Difference between the r^2 score (median across test runs) of the fine-tuned models (Conv1 to Conv7) minus baseline for each subject (sub-01 to sub-06) and the mean. Graphs are presented in the best predicted auditory (panel a) and visual (panel b) regions for the baseline model. STgyr_m: Superior Temporal gyrus middle; STgyr_p: Superior Temporal gyrus posterior; MTgyr_p: Middle Temporal gyrus posterior; HSgyr: Heschl's gyrus; VVISnet_l: Ventral Visual network lateral; PVISnet_l: Posterior Visual network lateral; LVISnet_DP: Lateral Visual network dorsoposterior.

Our main goal was to see if fine-tuning an auditory model improved brain encoding performance. We adjusted SoundNet at different depths, using our training data (seasons 1 to 3), then tested the model using season 4. We calculated the median r^2 score for each of the 210 parcels of the MIST ROI atlas and averaged the performance across all subjects (Figure 3).

The best-predicted ROIs by the baseline model showed a consistent pattern (Figure 3). Adjusting models at Conv7, Conv6, or Conv5 did not improve brain encoding and even worsened it for the STG middle. However, models adjusted at Conv1 to Conv4 significantly improved over the baseline, with Conv4 showing the most substantial improvements. Adjusting at Conv1 to Conv3 only gave slight or no improvements over Conv4 for most ROIs, except the left lateral posterior visual network. We decided to focus on Conv4 models for further experiments based on these results.

Supplemental File F - Consistency of parcels best predicted between subjects

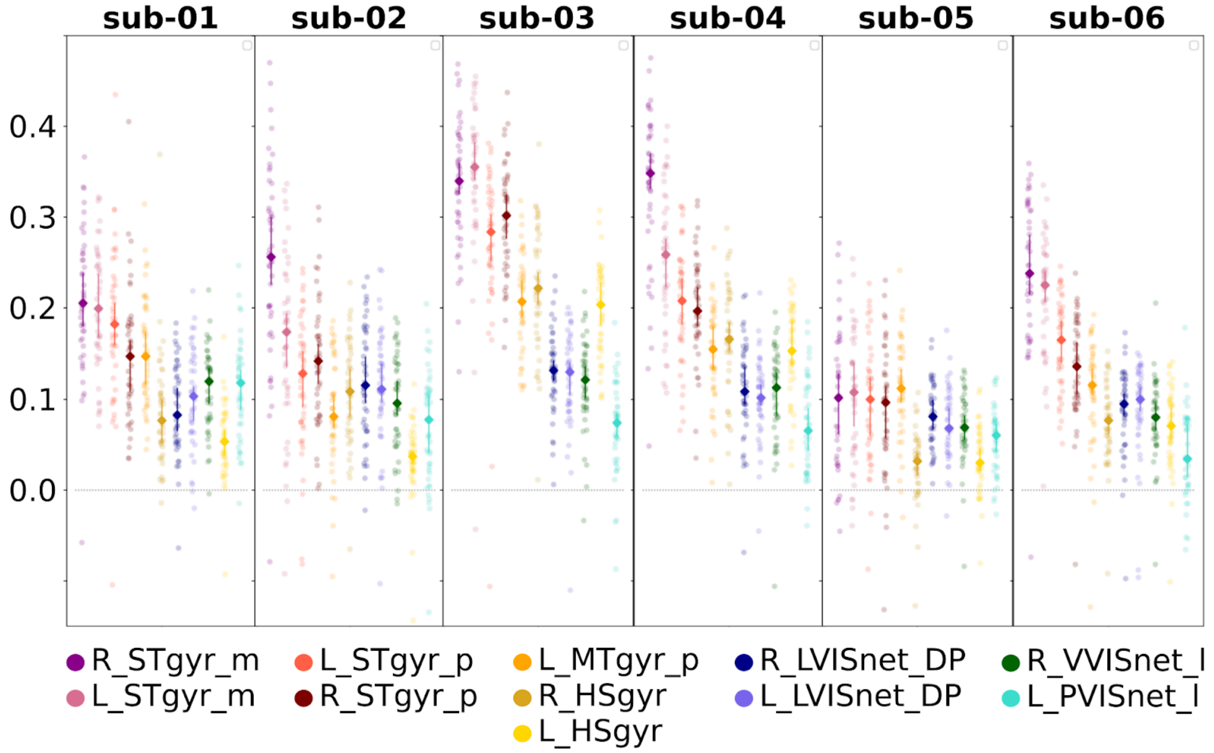

Figure S2. Distribution of r² scores across 48 runs (friends S04) for the eleven ROIs, with the highest average r² score across 6 subjects. The ROIs have been ordered by their median value across subjects and runs. The r² performance indicates the quality of prediction of fMRI time series in a single run (10 min. duration). R_STgyr_m: Right Superior Temporal gyrus middle; L_STgyr_m: Left Superior Temporal gyrus middle; L_STgyr_p: Left Superior Temporal gyrus posterior; R_STgyr_p: Right Superior Temporal gyrus posterior; L_MTgyr_p: Left Middle Temporal gyrus posterior; R_HSgyr: Right Heschl's gyrus; L_HSgyr: Left Heschl's gyrus; R_LVISnet_DP: Right Lateral Visual network dorsoposterior; L_LVISnet_DP: Left Lateral Visual network dorsoposterior; R_VVISnet_l: Right Ventral Visual network lateral; L_PVISnet_l: Left Posterior Visual network lateral.

Supplemental File G - Encoding brain activity at the voxel level without spatial smoothing

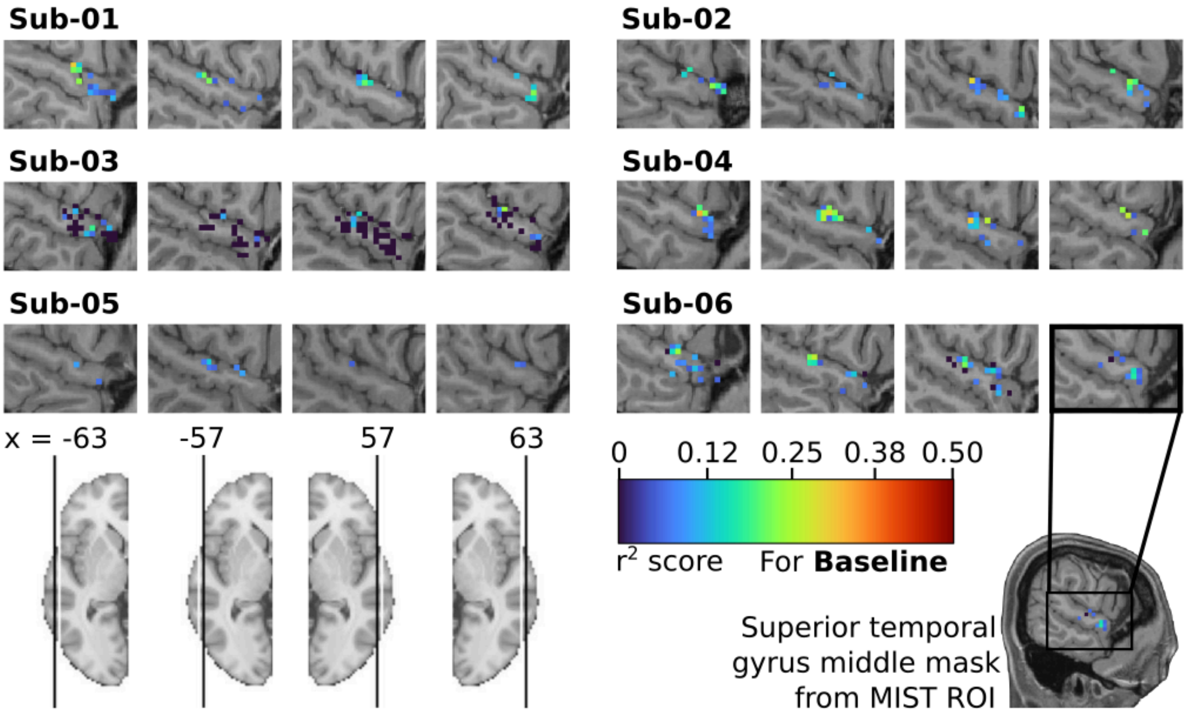

Figure S3. STG encoding using Soundnet with no fine-tuning and no spatial smoothing. Mapping of the r^2 scores from 556 voxels inside the cerebral region defined as the Middle STG by the parcellation MIST ROI, computed by the individual Baseline model. To have a better representation of the STG, 4 slices have been selected in each subject, 2 from the left hemisphere (-63 and -57) and 2 from the right hemisphere (63 and 57). Only voxels with r^2 values significantly higher than those of a null model initialised with random weights are shown (Wilcoxon test, FDR $q < 0.05$). Individual anatomical T1 have been used as background.

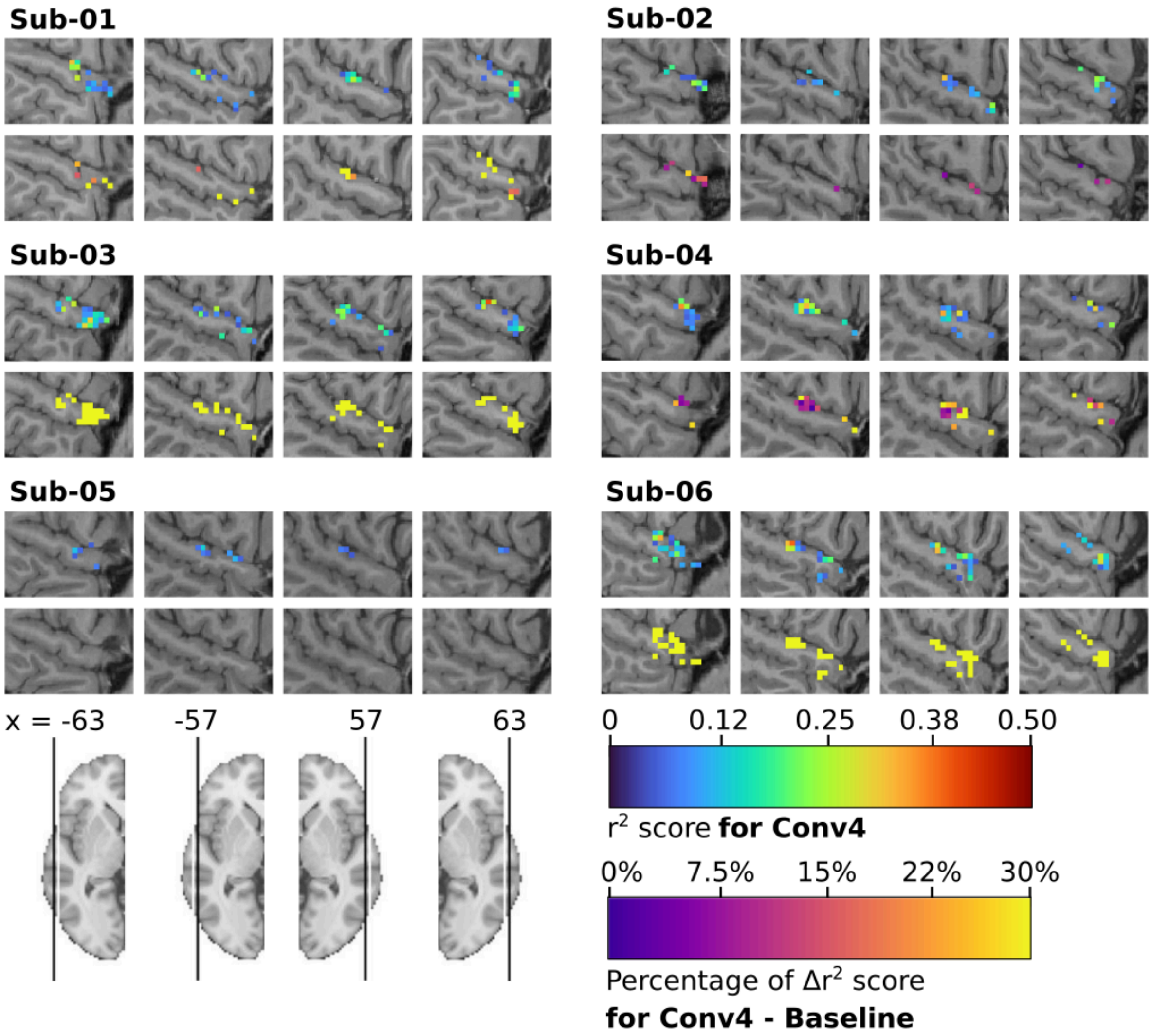

Figure S4. STG encoding using Brain-Aligned SoundNet and fMRI data with no spatial smoothing. For each subject, on the top : mapping of the r^2 scores from 556 voxels inside the cerebral region defined as the Middle STG by the parcellation MIST ROI, computed by the individual Conv4 model. Only voxels with r^2 values significantly higher than those of a null model initialised with random weights are shown (Wilcoxon test, FDR $q < 0.05$). For each subject, on the bottom : mapping of the difference of r^2 scores between the Conv4 model and the baseline model. Only voxels from the Conv4 model with r^2 values greater than +/- 0.05 and significantly greater or lesser than those of the baseline model are shown (Wilcoxon test, FDR $q < 0.05$). Individual anatomical T1 have been used as background.

Supplemental File H - Correlation between audio labels in Friends and change in prediction accuracy between individual baseline and brain-aligned models

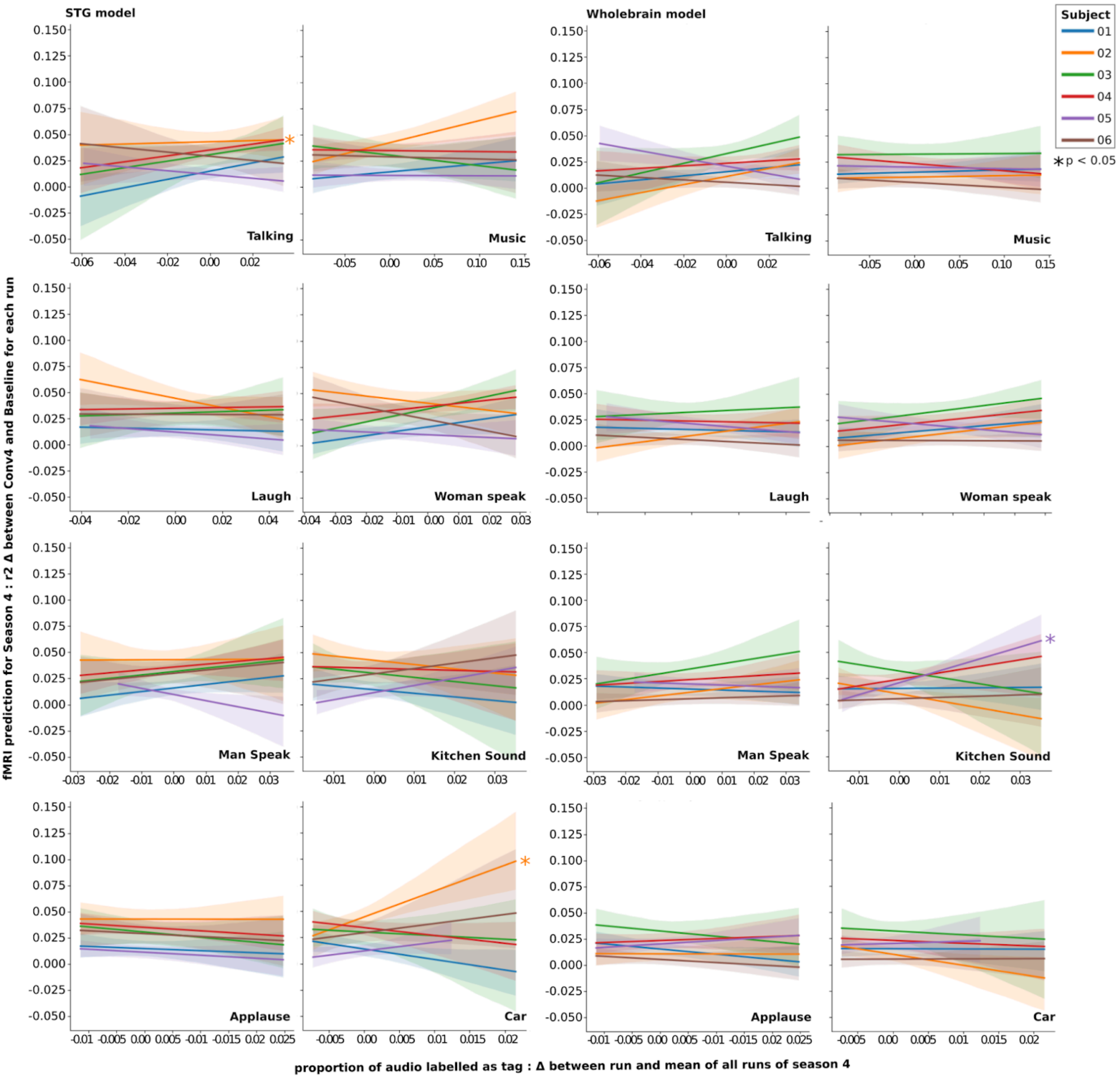

Figure S5. Linear regression of tagged audio proportion on prediction difference between brain-aligned and baseline model in season 4. Linear regression has been computed for each category, subject and model: We used the difference between the percentage of labelled audio of each half-episode and the mean percentage for all season 4 as the regressor, and the difference in max r² score between the baseline and brain-aligned models as the dependent variable. A multivariable regression (Ordinary Least Square) has also been computed for each subject and each model, using every category as regressors to explain the difference in max r² score. Significance (star) has been added to regressors with a significant ($p < 0.05$) in the OLS regression.

Supplemental File I - HEAR EVAL detailed score results

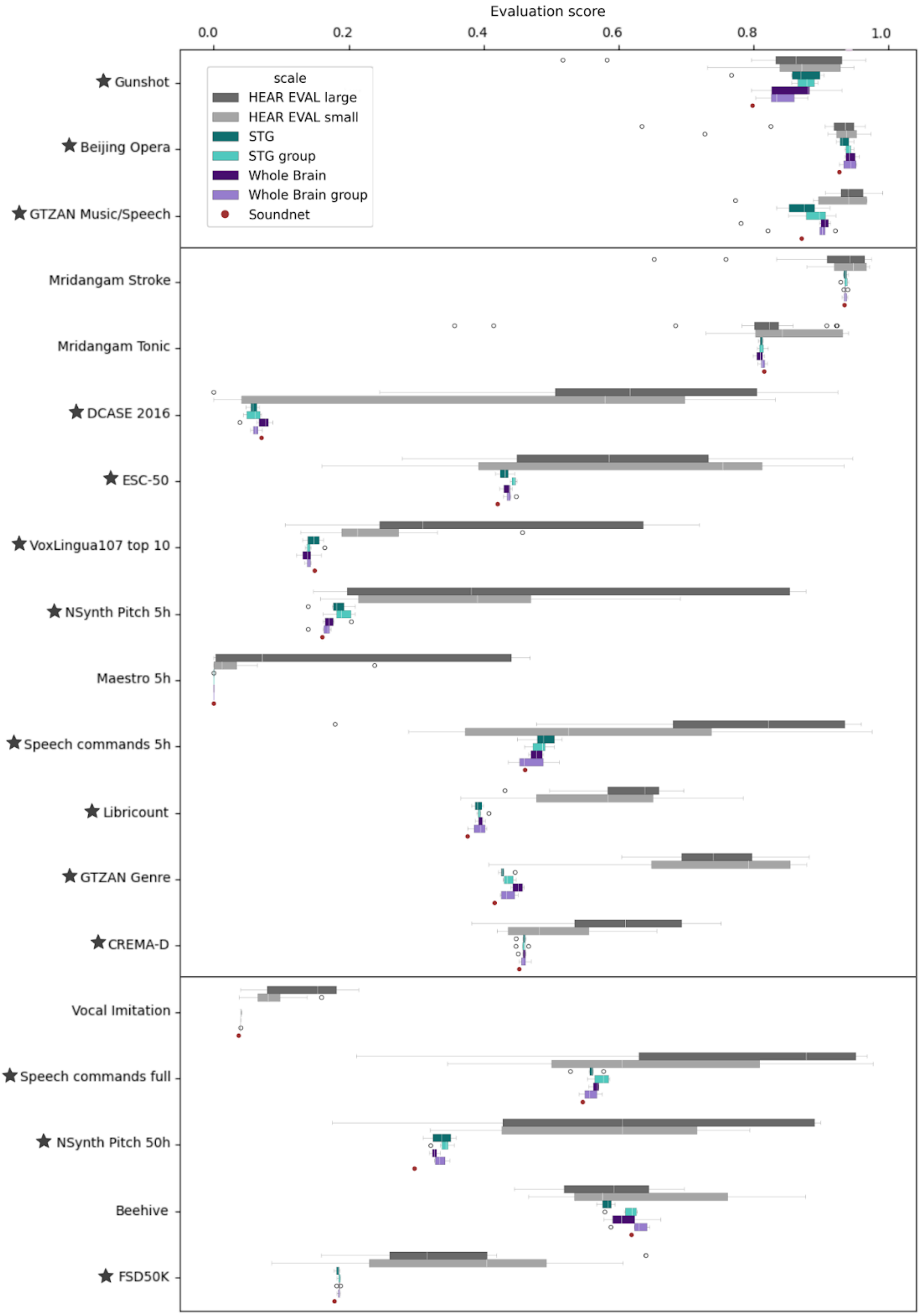

Figure S6. Distribution of all model's scores. The models are grouped into the following categories based on their characteristics: *HEAR EVAL large* for models from the HEAR benchmark with more than 20 million parameters (up to 1339M), *HEAR EVAL small* for models from the HEAR benchmark with fewer than 12 million parameters, *STG* for models that are brain-aligned with fMRI data from the Superior Temporal Gyrus (STG) of a single subject, *Whole Brain* for models that are brain-aligned with a parcellation of the entire brain from a single subject, and respectively, *STG group* and *Whole Brain group* when using fMRI data from five subjects. *SoundNet* serves as the baseline, showing the performance of the pretrained SoundNet model, which is not brain-aligned. Tasks are ordered by size of the dataset used for training (estimation based on the HEAR paper) and separated in three categories: Small dataset (inferior to 1h, between 1h and 10h, more than 10h). Different metrics have been used depending on the task (Accuracy for classification, pitch accuracy, Onset only F-measure, aucroc, mAP). For each metric, a higher score relates to a better performance. For tasks annotated with a star, brain-aligned models performance is significantly different from SoundNet (Wilcoxon test, $p < 0.05$). Brain-aligned models significantly degrade network performance in two tasks only, DCASE 2016 and VoxLingua 107 top 10. In all other annotated tasks, performance has been improved.

References

Behzadi, Y., Restom, K., Liau, J., & Liu, T. T. (2007). A component based noise correction method (CompCor) for BOLD and perfusion based fMRI. *Neuroimage*, *37*(1), 90-101.https://doi.org/10.1016/j.neuroimage.2007.04.042

Boyle, A. J., Pinsard, B., Borghesani, V., Paugam, F., DuPre, E., & Bellec, P. (2023, August 24). The Courtois NeuroMod project: quality assessment of the initial data release (2020) [Poster Abstract]. 2023 Cognitive Computation Neuroscience, Oxford, United Kingdom. https://2023.ccneuro.org/view_paper2f1e.html?PaperNum=1602

Esteban, O., Birman, D., Schaer, M., Koyejo, O. O., Poldrack, R. A., & Gorgolewski, K. J. (2017). MRIQC: Advancing the automatic prediction of image quality in MRI from unseen sites. PloS one, 12(9). https://doi.org/10.1371/journal.pone.0184661
